# A new Twitter based credit rating model methodology

**DOI:** 10.1007/s10479-026-07067-3

**Published:** 2026-03-04

**Authors:** Leonie Goldmann, Jonathan Crook, Raffaella Calabrese

**Affiliations:** Credit Research Centre, University of Edinburgh, Business School, 29 Bucceleuch Place, EH8 9JS Edinburgh, UK

**Keywords:** Risk Management, Credit Ratings, Forecasting, Social Media Data

## Abstract

In this paper we propose a novel way to predict corporate credit ratings by showing how a new type of data, Twitter, can be extracted and used for this purpose. We make three contributions to knowledge. First, we relate tweets from the companies themselves and tweets about the companies to the probability of a credit rating level. Second, we transform the tweets into two different sentiment scores which are used as predictors for credit rating levels and compare their predictive performance. The sentiment scores are calculated by using each of two alternative word-lists. Third, we propose two approaches how alternative information from Twitter, linguistic features in tweets, can be selected and incorporated into credit rating models. We compare the performance between the models containing the sentiment scores and the linguistic features. We analyze data relating to NASDAQ and NYSE listed companies over 2011-2019. Overall, we find that including information from Twitter gives a higher predictive performance compared to those of models that omit them.

## Introduction

Over the last ten years the number of social media users has increased dramatically. For example, according to Statista^1^ the number of monthly active Twitter users increased from approximately 30 million in 2010 to around 370 million in 2022. Each social media platform has a different objective, but what most of them have in common is that they constantly generate massive amounts of textual data. Analyzing this type of data in order to discover unknown structures has become a popular research stream among scientists from all types of fields, such as medicine (e.g. (Schmidt, 2012)), politics (e.g. Cameron et al. (2016)) and finance (e.g. Teplova et al. (2023)).

This paper analyzes social media data from and about large listed US companies. More specifically, we show how information from Twitter can be extracted and used to predict corporate credit rating levels of large US companies. In particular, we analyze whether information from Twitter can be used to predict credit rating levels and we show the effects of using different sources of tweets and information contained in them. Predicting corporate credit rating levels has been the focus of a large number of studies (e.g. Hwang et al., 2009 and Yuan et al., 2018), because the methods used by rating agencies to assign credit ratings are not publicly revealed. Credit ratings are an independent measure of how likely a company is to service its debt obligations and can be interpreted as a measure of a company’s riskiness in this sense. Therefore, they are important to a variety of different entities, such as investors, rating agencies and the companies themselves.

We are relating social media data to corporate credit rating levels since this type of data conveys information that is not enclosed in conventional predictors. We use information from Twitter and not any other social media platform since it is one of the largest social media platforms by user numbers and since the data is publicly available. Additionally, Twitter data has been used for predictive modeling in many different domains before, and especially within financial applications it has shown great results (e.g. Bollen et al., 2011 and Yuan et al., 2018). We analyze tweets from and about companies, since both Twitter sources reflect different aspects of the companies that could be predictive of credit rating levels. Tweets from the companies themselves are information the companies strategically publish and hence give internal information about the companies and their behavior. Tweets about the companies are expressed by externals, and can therefore be interpreted as the public’s opinion about the companies.

This paper adds to the existing literature in two ways. First, we relate tweets from the companies themselves to corporate credit rating levels, which to our knowledge has not been done before. Tweets from the companies themselves are a proxy for a company’s marketing strategy and can reflect strategic communication efforts aimed at shaping public perception, managing investor expectations, or signaling transparency and confidence (e.g. Jung et al. (2018)). Therefore, they could be predictive of credit rating levels.

Second, the information we extract from the tweets and the resulting predictors have not been used to predict corporate credit rating levels before. More specifically, we relate the tweet frequency and two different sentiment scores based on positive and negative word-lists to the credit rating levels as well as selected linguistic features contained in tweets. Sentiment scores have the advantage that they include all the information contained in the tweets and give an overall indication of whether a tweet is positive or negative. However, they rely heavily on the selected word-list. Hence, as an alternative approach we select specific linguistic features and incorporate these as additional features into the model. For this, we first apply differential language analysis (DLA) (Schwartz et al., 2017) to show which linguistic features contained in tweets from and about companies are correlated with corporate credit rating levels. We then show how the identified linguistic features can be selected and used for corporate credit rating level prediction. Differential language analysis has not been applied within a financial context before and has the advantage that it does not rely on an a priori fixed set of words. Additionally, the predictive n-grams reveal more subtle linguistic cues, such as framing choices, repeated phrases, or tone shifts, that are not captured by traditional sentiment dictionaries. We compare the models containing the different Twitter predictors and analyze their performance.

In addition to these contributions to the literature, we use a unique annual data-set comprising around 2000 observations on more than 300 different companies. This data-set contains tweets from and about corporate Twitter accounts, S&P credit ratings and four financial ratios that have primarily been used in this context before. We compare the performance of models with and without Twitter variables for predicting credit rating levels. Using a random forest classifier we show that including information from Twitter increases predictive performance for almost all model specifications. We further find that different sentiment scores lead to different results and that therefore the choice of the word-list when calculating the sentiment score is crucial. Last, we find that using linguistic features contained in tweets as additional predictors can lead to superior performance as opposed to using sentiment scores and the tweet frequency.

This paper is structured as follows: Section 2 is a summary of the relevant literature. Section 3 outlines the methodology, more specifically random forest, sentiment analysis and differential language analysis. Section 4 describes the Twitter and credit rating data that we use and the main empirical results. Finally, Section 5 presents the conclusion of the research and possible extensions of this paper.

## Literature Review

Because rating agencies do not fully disclose the models underlying their rating decisions, a substantial body of research has emerged that aims to predict credit ratings using observable firm-level information. In the following, we organize this literature along two main dimensions: the empirical methods applied and the types of input variables considered.

The existing literature on credit rating prediction employs both traditional statistical approaches and machine learning techniques. Commonly used statistical methods include ordered probit models (e.g. Hwang et al., 2009), ordinal logistic regression (e.g. Hwang et al., 2009 and Yuan et al., 2018), and multiple discriminant analysis (e.g. Lee, 2007). In addition, a range of machine learning methods has been applied, such as neural networks (e.g. Kumar & Bhattacharya, 2006), support vector machines (e.g. Lee, 2007) and random forests (e.g. Yuan et al., 2018). Overall, prior studies indicate that machine learning approaches, particularly random forests, tend to achieve higher predictive accuracy than standard statistical methods (Yuan et al., 2018). Accordingly, this paper adopts a random forest classifier to predict credit rating levels.

The selection of variables commonly associated with corporate credit ratings is largely informed by the closely related bankruptcy prediction literature. Although a wide range of input variables has been considered in prior bankruptcy studies, accounting-based measures expressed as financial ratios are generally identified as the most important determinants of bankruptcy risk (Beaver, 1966; Altman, 1968 and Ohlson, 1980). A prominent example is the Z-score introduced by Altman (1968), one of the most widely used bankruptcy prediction models in the literature (Niresh & Pratheepan, 2015; Altman et al., 2013; Jackson & Wood, 2013 and Almamy et al., 2016), which combines four or five financial ratios in a linear framework. Beyond financial ratios, previous studies have also incorporated market-based variables (Merton, 1974; Bharath & Shumway, 2004 and Hillegeist et al., 2004) as well as macroeconomic variables (Hernandez Tinoco & Wilson, 2013; Nam et al., 2008 and Qu, 2008).

Similarly to the bankruptcy prediction literature, the variables traditionally used for corporate credit rating prediction and widely accepted among researchers are accounting variables, such as a company’s retained earnings and working capital (Kim & Sohn, 2008). Some studies also include market-based variables, such as the stock price (Hájek & Michalak, 2013) or macroeconomic variables, such as the unemployment rate and GDP growth rate (Kim & Sohn, 2008).

Accounting variables are typically obtained from a company’s quarterly or annual reports and are commonly transformed into financial ratios motivated by the Z-score framework (Kim & Sohn, 2008). These measures are intended to capture a company’s overall financial condition (Kim & Sohn, 2008) and are therefore widely used as predictors of credit rating levels. There are several additional reasons for their frequent use in corporate credit rating prediction. First, such variables are readily available from publicly accessible financial statements. Second, they are standard inputs in corporate bankruptcy prediction studies (Altman, 1968), which are closely related to the credit rating literature (Matthies, 2013). Third, prior empirical evidence shows that commonly employed accounting variables are statistically significant and informative for predicting corporate credit ratings (see Kim & Sohn, 2008 and Hájek & Michalak 2013). Fourth, these variables capture key dimensions of a company’s creditworthiness, such as leverage, firm size, and profitability, and thus have a direct relationship with corporate credit ratings.

Market-based variables capture how a company’s past performance and future prospects are reflected in market assessments (Hájek & Michalak, 2013) and typically include measures such as the stock price and the high/low stock price. Compared to accounting variables, market-based indicators are less frequently employed in credit rating studies; however, several authors have documented their relevance for explaining corporate credit ratings. For instance, (Hájek & Michalak, 2013) performed a variable selection analysis for credit rating prediction and found that a range of different algorithms almost always selected the stock price and the high/low stock price as important predictors. From a theoretical perspective, the association between market-based variables and corporate credit ratings may be attributed to their role as proxies for firm value, with higher-valued companies expected to exhibit greater creditworthiness. At the same time, the existing literature applying market-based variables to credit rating prediction remains limited, and the precise impact of these variables on corporate credit ratings warrants further investigation.

Macroeconomic variables have also been considered as predictors of credit rating levels (see for example Kim & Sohn, 2008). As these variables are widely used in bankruptcy prediction studies (see Hernandez Tinoco & Wilson, 2013), their inclusion in credit rating analyses is motivated by the close relationship between bankruptcy risk and credit risk. Amato and Furfine (2004) further argue that macroeconomic conditions should be incorporated, as a company’s creditworthiness is expected to decline during economic downturns. While several studies include macroeconomic variables in credit rating prediction (e.g. Kim and Sohn, 2008; Amato and Furfine, 2004), the empirical evidence on their impact is mixed. Kim and Sohn (2008) show that exchange rates, consumer price inflation, changes in external debt, as well as other indicators such as the discount rate, unemployment rate, and GDP growth are not statistically significant. Moreover, rating agencies state that credit ratings are assigned on a through-the-cycle basis; consequently, issued ratings should, in theory, be largely independent of shortterm macroeconomic fluctuations.

Taken together, the literature indicates that accounting variables constitute the most robust and widely accepted predictors of corporate credit rating levels. In contrast, evidence on the incremental value of market-based and macroeconomic variables is less conclusive. Consequently, we focus on accounting variables as core control variables in our empirical analysis.

Recently, there have been studies that show how novel data sources can be used for credit rating predictions, such as social media data (Yuan et al., 2018), qualitative information from annual reports (Hájek et al., 2017), the CEOs’ birth order (Park et al., 2022) and many others. The use of variables from novel data sources is motivated by previous studies that showed how these variables can successfully be used to predict related financial variables, such as stock returns (Bollen et al., 2011; Figa-Talamanca & Patacca, 2022 and Oliveira et al., 2013), bankruptcy or default (Cecchini et al., 2010; Zhao et al., 2019 and Putra, 2018) and credit default swap spreads (González-Fernández & González-Velasco, 2020; Yang et al., 2020 and Liebmann et al., 2016). Since stock returns relate to a company’s performance which is closely related to a company’s creditworthiness and since bankruptcy and credit default swap spreads directly relate to a company’s credit risk, it is intuitive to include this information in corporate credit rating studies as well.

The study by Yuan et al. (2018) is, to our knowledge, the only study where information from Twitter has been used for corporate credit rating prediction. While our research is similar in its overall aim, it diverges in two important and novel ways. First, rather than relying solely on tweets about companies, we also incorporate tweets by the companies themselves. This allows us to capture aspects of corporate communication strategy, such as tone, framing, and consistency, which may provide soft signals relevant to creditworthiness. This may be important to predictive accuracy if, for example, there is a sudden change in the future prospects of a firm that is anticipated by management but not by external commentators. Asymmetric information between managers and outsiders is extremely prevalent, hence predictions based on statements from management communications may be more immediately predictive than merely comments by external actors. Second, we adopt a very different method of extracting latent information from tweets. Unlike Yuan et al. (2018), who matched words to distributions of eight ’emotions’, we extract the most predictive n-grams from company-related tweets using differential language analysis. This enables us to move beyond coarse positive/negative sentiment to capture richer linguistic patterns that may convey nuance, emphasis, or domain-specific terminology. Together, these extensions allow us to evaluate not only the informational content of social media discourse but also how a company’s own use of language contributes to credit rating prediction. For papers that discuss the usage of Twitter data for predictions in related contexts, see the recent literature review by Cano-Marin et al. (2023).

## Methodology

In this paper we use a random forest classifier to predict corporate credit rating levels, since it has shown good results in the existing literature (e.g. Yuan et al., 2018). Furthermore, we use sentiment analysis to transform the raw Twitter data into variables that can be included as predictors in the model and differential language analysis to find the linguistic features in tweets that are most predictive of specific corporate credit rating levels. While these methods may appear simpler compared to state-of-the-art NLP techniques such as large language models (LLMs), they offer distinct advantages in terms of computational efficiency, transparency, and domain relevance, particularly in high-stakes fields like finance and highly regulated sectors like banking, where interpretability is essential. Sentiment scores derived from dictionaries can be directly traced back to specific words, offering a clear and immediate explanation for their values. In contrast, while interpretability techniques such as attention maps (e.g., in transformer models like BERT) can provide insights into model behavior (Wiegreffe & Pinter, 2019), these are post-hoc and often less intuitive, especially given the multi-headed and layered structure of such models. Attention does not always equate to explanation (e.g. Wiegreffe & Pinter, 2019), and interpreting these mechanisms remains an ongoing area of research (Liu et al., 2022, Neely et al., 2022 and Bussotti & Papotti, 2025).

Moreover, we do observe predictive uplift from these linguistically derived features which suggests that more complex NLP architectures may not be necessary for this task. While lightweight transformer-based models could potentially be applied, our goal is not to outperform black-box approaches but to assess the incremental predictive value of Twitter features. Finally, beyond the issue of interpretability, the computational demands of even moderately sized LLMs can be substantial at inference time, whereas our approach requires minimal resources, making it more practical and scalable for integration into real-world financial modeling pipelines.

### Random forest

**Fig. 1:**
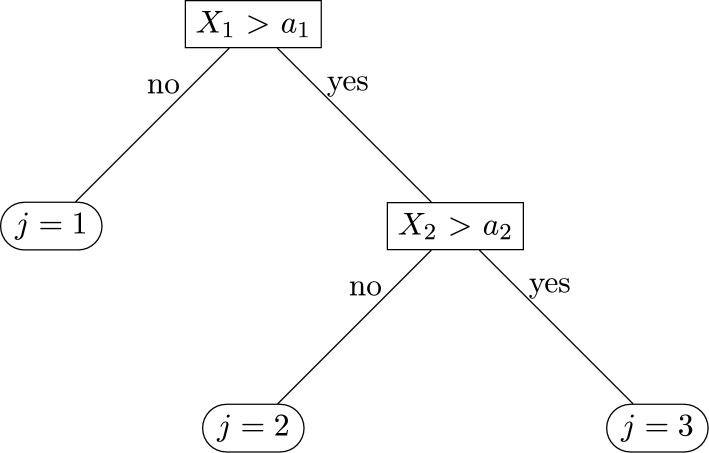
Example of a simple decision tree with two features and three outcome categories

A random forest is a classification algorithm, more specifically an ensemble-learning algorithm that generates many tree predictors and aggregates the results (Breiman, 2001). Each tree predictor depends on a generated random vector and the values of these random vectors are independently sampled but follow the same distribution. For classification, each of these trees gives a decision and the class with the most votes determines the overall classification. Each tree consists of nodes which test the value of a certain attribute, branches which correspond to the outcome of a test and connect to the next node or leaf and leaf nodes which are the terminal node that predict the outcome. An example of such a decision tree for classification is shown in Figure 1. In this example we show a simple decision tree for classification with three categories $j=1,2,3$. Assuming we have $i=1,..,n$ observations and covariates $\textbf{X}=X_1,X_2$ with outcomes $Y_1,Y_2,Y_3$. Each node has a binary decision based on whether $X_1>a_1$ and $X_2>a_2$ or not for fixed $a_1$ and $a_2$. The parameters $a_1$ and $a_2$ are therefore the decision parameters and $X_1$ and $X_2$ are the two features.

The node on the top contains all observations which are subdivided among the branches according to the classification at that node. In our example this corresponds to $X_1>a_1$. This subdivision continues until all leaf nodes have observations of only one category. The decision parameters $a_1$ and $a_2$ and the features $X_1$ and $X_2$ are chosen so that each following node is as pure as possible, which means not having a lot of diversity in terms of categories $Y_j$. The diversity can be measured in different ways. The impurity function used in this analysis is based on the Gini Index, which is defined as:

1 $$\begin{aligned} i_G(t)= \sum _{j=1}^{3}p(j|t)(1-p(j|t))=1-\sum _{j=1}^{3}p(j|t)^2 \end{aligned}$$

where *t* is the node where the split is performed and *p*(*j*|*t*) the probability of randomly picking an element of category *j* at this node. Therefore, the Gini based impurity function measures how often a randomly chosen object would be classified incorrectly when randomly labeling it according to the class distribution *p*(*y*|*t*). It can be seen as a criterion to minimize the probability of misclassification. More information on decision trees can be found in Safavian and Landgrebe (1991). Notice that in simple trees, each node is usually split using the best split among all variables (Friedl & Brodley, 1997). In a random forest, each node is split using the best among a subset of predictors ($m_{try}$) randomly chosen at that node. This strategy is used, since it is robust against overfitting and it has performed well compared to other non-linear classifiers (Liaw & Wiener, 2002). It is also highly non-linear and allows for interactions between the predictors. A more detailed description of the random forest algorithm can be found in Breiman (2001).

For estimating the random forest classifier we use the ’randomForest’ **R** package^2^, which is based on Breiman (2001). We specify $n_{tree}=1000$ in order to ensure that every input row gets predicted at least a few times and we choose the optimal value of predictors $m_{try}$ by using the **R** function *tuneRF*. This function returns the optimal $m_{try}$ parameter with respect to Out-of-Bag error estimates. Out-of-Bag error estimates are errors estimates for observations that have not been used for training the algorithm. For calculating the Out-of-Bag error estimates for different values of $m_{try}$ the random forest algorithm is trained on a random subset of the training data and predictions are made on the observations that were not used for training the algorithm. Recent applications of random forests to corporate risk include García et al. (2019) and Uddin et al. (2020) among others.

### Sentiment Analysis

Sentiment analysis is the activity of classifying text into different types of sentiments, for example into positive, negative or neutral sentiments. This is often done in order to find out a writer’s attitude towards a particular topic or to find out a writer’s general mood. We use sentiment analysis in order to transform the raw Twitter text into sentiment scores that can be included as an explanatory variable into our models. In order to calculate such a sentiment score we use a lexicon-based approach, that is based on a word-list. The list of words has predetermined emotional directions (i.e. positive or negative) and text is classified into its sentiment according to the occurrences of these words in the text.

Before we start with counting the occurrences, we first clean up the text by removing digits, punctuation characters and control characters. Next, we convert the text into lower case and we split it into single words. Last, we apply an algorithm that counts the matches of positive words and negative words for each text and subsequently calculates a score of $N_{pos}-N_{neg}$ where $N_{pos}$ is the number of occurrences of positive words and $N_{neg}$ is the number of occurrences of negative words. This sentiment score gives a good indication of how positive or negative the text is. Since we only count the positive and negative words according to the word-lists, it is not necessary to remove stop words or URLs.

The sentiment score is calculated for each tweet and, if multiple tweets are considered (i.e. all tweets of one day that mention a specific company), many authors calculate some type of average and use it for prediction (see for example Kearney and Liu (2014) or Ranco et al. (2015)). Our approach differs from this standard method, as we do not calculate a score for each tweet within the year and take the average, but instead treat all tweets of one year together and calculate one overall sentiment score. We think that this approach is more sensible for the prediction of long-term credit ratings, since it reflects the sentiment of tweets about or from companies without the risk of the score decreasing due to uninformative tweets. For example, tweets from companies themselves are often advertisements that are not likely to contain many negative words. Hence, averaging over such “uninformative” tweets would lead to a decreased sentiment score that might not reflect the annual sentiment of the tweets appropriately. We also expect that uninformative tweets are ignored by the public, and only tweets that are informative and have a strong sentiment are perceived, which further strengthens our approach of not averaging over the number of tweets.

We choose two different word-lists for the sentiment score calculation, both containing positive and negative words. The first word-list is from the opinion lexicon^3^ of Hu and Liu (2004). We choose this word-list since it has been created specifically for social media texts and has shown good results in a finance application before (Si et al., 2013). The list of positive and negative words has been compiled by the authors over many years, starting from their first paper “Mining and summarizing customer reviews” (Hu & Liu, 2004). We refer to this word-list as the “SM-Word-list” (Social Media Wordlist). Similarly, we refer to the sentiment score that we calculate using this list as “SM score”. The second word-list was created by Loughran and Mcdonald (2011) and contains finance specific positive and negative words. For creating the word-list the authors first developed a dictionary of words and word counts from a large number of 10-Ks filing reports during 1994 to 2008. Only words that occur in at least 5% of the reports have been considered and carefully examined regarding their most likely usage. More details on how the word-list has been created can be found in Loughran and Mcdonald (2011). We choose this word-list as a comparison to the first one, since the authors showed that general word-lists often contain positive or negative words that are typically not positive or negative in a financial context, which could lead to biased results. We refer to this word-list as the “Finance-Word-list” and we refer to the sentiment score that is calculated using this list as “Finance score”. Since the words ’upgrade’ and ’downgrade’ are typically used for a positive or negative change, respectively, in credit rating we add these words to both word-lists.

Such lexicon-based approaches have shown great results in the literature, especially when considering that no training data-sets are needed (e.g. Fernández-Gavilanes et al., 2016). A disadvantage is that these algorithms rely heavily on the created or chosen word-list. For instance, simple lexicon-based approaches cannot detect negations, and if the chosen word-list is in some sense incomplete, not all positive or negative words can be detected. This may result in an incorrect classification of the text (Kolchyna et al., 2015).

### Differential language analysis

Differential language analysis (DLA) is an open-vocabulary approach that enables the identification of linguistic features in language which are individually most predictive of a discrete outcome or that independently explain the most variance for continuous outcomes (Schwartz et al., 2017). DLA is implemented in the differential language analysis toolkit (DLATK) (Schwartz et al., 2017) that supports a number of different statistical algorithms for performing DLA^4^, such as linear regression, logistic regression or Pearson correlation among others.

Before applying DLA we tokenize the given sample of text in order to extract the linguistic features. Tokenizing means splitting the sentences into tokens, which include words, punctuation, emoticons as well as phrases. We refer to these linguistic features as n-grams, which are adjoining sequences of *n* elements from a given sample of text. Hence, a 1-gram is a single element (e.g. a word or a smiley), a 2-gram are 2 elements following each other and a 3-gram are 3 elements following each other. We use 2-grams and 3-grams in addition to 1-grams since groups of elements taken together can often give more insight into our extra linguistic information than a single element. The emoticon-aware tokenizer we use was built by Schwartz et al. (2013) and is based on Christopher Pott’s^5^ “happyfuntokenizer” that has been extended in order to allow it to capture emoticons like $<3$ (a heart) or :-) (a smile).

In the next step, we normalize the n-gram counts by the total n-gram use of each group:

2 $$\begin{aligned} \frac{freq(\text {n-gram},\text {group})}{freq(*,\text {group})}. \end{aligned}$$

The term in the numerator $freq(\text {n-gram},\text {group})$ denotes the frequency of one n-gram in a group and the term in the denominator $freq(*,\text {group})$ denotes the frequency of all n-grams in a group. This is done in order to get the proportion of each n-gram in the group. The definition of the group depends on the dependent variable of the specific application. As we are interested in linguistic features that are predictive of annual credit ratings, the tweets of a company’s year form the groups. In other words, all tweets that relate to one observation of the dependent variable (company A in year 2015) form one group. This holds for tweets from companies as well as for tweets about companies. In the existing literature the dependent variables are mostly sociological or psychological variables of one individual. Therefore, the groups are mostly defined by Twitter user (see for example Pang et al., 2019).

Before using the normalized frequencies of words and phrases as predictors for our independent variables we eliminate uninformative phrases (2-grams and 3-grams). We do this by applying a collocation filter based on point-wise mutual information, denoted as *pmi* (Church & Hanks, 1990) on each phrase. This is necessary in order to determine the difference between the independent probability of a token and the joint-probability of two or three successive tokens occurring together in a 2-gram or 3-gram (Schwartz et al., 2013a):

3 $$\begin{aligned} pmi(\text {phrase}) = log \frac{p(\text {phrase})}{\prod _{\text {token} \in \text {phrase}} p(\text {token})} \end{aligned}$$

In this equation $pmi(\text {phrase})$ denotes the point-wise mutual information of the phrase. $p(\text {phrase})$ is the probability of the phrase in the text, which is the joint probability of the tokens of that phrase, and $\prod _{\text {token} \in \text {phrase}} p(\text {token})$ is the product of the individual probabilities of observing the tokens contained in the phrase. A high value of $pmi(\text {phrase})$ represents a phrase that consists of tokens whose probability of co-occurrence is similar to the individual probabilities of the tokens. A low value of $pmi(\text {phrase})$ represents a phrase that consists of tokens whose probability of co-occurrence is considerably lower as opposed to the individual probabilities of the tokens. In order to ensure that only phrases are kept that are informative parts of speech, we eliminate phrases with point-wise mutual information smaller than 2. As 3-grams have higher *pmi* values than 2-grams we first normalize the *pmi* values by dividing the *pmi* by the number of words minus one. More information can be found in Bouma (2009). Additionally, we exclude linguistic features that are used by less than 1% of groups in order to avoid high dimensional issues and in order to prevent overfitting (Zamani et al., 2018).

After these pre-processing steps, we apply DLA by relating each remaining normalized n-gram to our dependent variable. For a binary outcome we do this by using logistic regression models. Since many features are explored at once^6^, we need to control the false discovery rate, which is done by using the Benjamini-Hochberg procedure (Benjamini & Hochberg, 1995) at level $\alpha =0.05$ (Ferreira & Zwinderman, 2006). The Benjamini-Hochberg Procedure can be explained as follows (Thissen et al., 2002):

- The p-values of each feature are ordered in ascending order and ranked.
- A Benjamini-Hochberg critical value is calculated for each p-value, using the p-value’s rank *r*, the total number of features *f* and $\alpha =0.05$, so that $\frac{r}{f}*\alpha$.
- The feature with the highest p-value, that is also smaller than its corresponding Benjamini-Hochberg critical value, is considered significant as are all features with lower p-values.

A more detailed explanation of the methodology can be found in Schwartz et al. (2013a). Since analyzing thousands of linguistic features can result in many being statistically significant, it is helpful to visualize the results appropriately. The DLATK offers a visualization in the form of n-gram clouds images. These n-gram clouds images, also called word-clouds, depict statistically significant linguistic features in terms of relative correlation strength or marginal effect and relative frequency in the corpus^7^. The coefficient that reflects the correlation strength or effect of each n-gram depends on the statistical model that has been used for estimating the relationship between each n-gram and the dependent variable. For example when the Pearson correlation is used for estimating the relationship between each n-gram and a continuous variable, the n-grams are represented in the word-clouds in terms of their Pearson correlation coefficient (Pearson’s *r*). When using linear or logistic regression^8^ for performing DLA the n-grams are represented in terms of their $\beta$ regression coefficients^9^.

We note that the majority of the existing literature on DLA (Schwartz et al., 2017) does not only relate to the Pearson correlation coefficient as correlation strength, but also to the $\beta$-coefficients. We will refer to the $\beta$-coefficients of the n-grams as marginal effects in the remainder of this thesis, as this is a more accurate description. In a logistic regression framework the $\beta$-coefficients are generally described as the marginal effects with respect to the logits.

Visualizing the n-grams in terms of correlation strength or marginal effect and in terms of frequency in a word-cloud enables a better perception of the key results compared to simply displaying the large number of significant linguistic features and their key properties in a table.

The representation in terms of Pearson correlation/marginal effects and frequency is sensible when applying DLA to continuous outcomes using linear regression, but it is not a sensible representation of logistic regression $\beta$-coefficients, as $\beta$-coefficients of different logistic regression models cannot be compared. This is due to the unknown scaling because the dependent variable is assumed to be latent. See for more details the paper by Allison (1999). Therefore, we will not visualize our results using DLATK but instead propose another visualization that is particularly suitable for results of logistic regression models. Instead of displaying the Benjamini-Hochberg corrected significant n-grams in terms of frequency and $\beta$-coefficient we display these n-grams in terms of frequency and p-value. We do this, as p-values of different logistic regression models can be compared and are therefore a more informative comparison for this application as opposed to the $\beta$-coefficients.

## Empirical Analysis

In this section we outline the data collection process and analyze the data descriptively. Then, we apply the methodology to the data and discuss the results.

### Data

Our empirical analysis focuses on companies listed on the New York Stock Exchange^10^ (NYSE) and NASDAQ^11^. Credit rating and financial statement data are obtained from Thomson Reuters Eikon and Datastream (Refinitiv), while Twitter data are collected via Twitter’s official API^12^.

#### Dependent variable

The dependent variable is a company’s grouped S&P credit rating level, as summarized in Table 1. S&P assigns long-term issuer ratings ranging from AAA to D, with intermediate categories distinguished by plus and minus notches^13^. To ensure a sufficient number of observations per category, we collapse these notches and aggregate ratings into five broader groups, “Over AA”, “A”, “BBB”, “BB”, “Below B”, following common practice in the literature (e.g., Kim and Sohn 2008). Table 1

**Table 1:** Grouping method of S&P credit ratings

**Grouped Credit Rating**	**Credit Rating**
Over AA	AAA
	AA+
	AA
	AA-
A	A+
	A
	A-
BBB	BBB+
	BBB
	BBB-
BB	BB+
	BB
	BB-
Below B	B+
	B
	B-
	CCC+
	CCC
	CCC-
	CC
	C
	D

Because credit ratings are intended to reflect a company’s long-term creditworthiness, rating changes tend to occur infrequently. Consistent with prior studies, we therefore construct an annual dataset (e.g., Amato & Furfine, 2004 and Blume et al., 1998). For each year, we assign the rating observed at the end of the calendar year to the corresponding firm-year observation.

#### Financial variables

In line with the existing literature (e.g. Kim & Sohn, 2008; Altman & Rijken, 2004; Doumpos et al., 2015 and Altman, 1968), we include annual financial variables as control variables. Specifically, we collect net working capital (WC), total assets (TA), retained earnings (RE), book value of total liabilities (BL), earnings before interest and taxes (EBIT), and market value of equity (MV), and construct the ratios WC/TA, RE/TA, EBIT/TA, and MV/BL. The variables are all obtained annually from Refinitiv^14^. Since the market value of equity *MV* is defined as the share price multiplied by the number of ordinary shares in issue it is not in fact an annual variable that can be obtained from a company’s annual balance sheet, such as the working capital, but is a daily variable. Therefore, we need to transform the market value into an annual variable in order to ensure consistency among the financial variables. Aligning with the credit rating levels we use the market value of a company’s last day of the year as reference point.

We choose these ratios because they have been commonly used in the existing literature and because they are publicly available and hence we expect that rating agencies as well as externals take them into account when evaluating the companies. We also include them because there are strong a priori arguments as to why they would be predictive of credit ratings. We limit our analysis to four financial ratios^15^ as control variables. Huang et al. (2004) showed that using a small subset of financial indicators can achieve predictive performance comparable to, or even exceeding, models that include larger sets of indicators. This approach of focusing on a few key financial variables is also common in the credit rating literature: for instance, Kim and Sohn (2008) and Altman and Rijken (2004) each employed four financial indicators, while Yuan et al. (2018) used five. Moreover, credit rating prediction is closely linked to bankruptcy prediction, where one of the most widely known models, the Altman Z-score, relies on only four or five financial ratios (Yuan et al., 2018). We do not include additional control variables such as macroeconomic indicators or corporate governance measures. The inclusion of governance variables is beyond the scope of this study, and rating agencies emphasize a through-the-cycle approach, suggesting that short-term macroeconomic fluctuations should have limited influence on assigned credit ratings.

Even though we would expect that the lagged credit rating would be a strong predictor of credit ratings, we do not include the lagged credit rating as a predictor for two reasons. First, we want to build a model that can be used for the prediction of credit ratings of companies that have not been rated before. Second, we are interested in the predictive power of the Twitter variables. If we would use the lagged credit rating level as a predictor, because of the rarity of changes in credit rating, the ratings would be predicted close to perfectly. Hence, when adding Twitter variables, even if they are powerful predictors, they may only marginally increase predictive accuracy since there would be little additional accuracy available to be gained. Additionally, we are interested in the determinants of a credit rating and therefore do not want to build an autoregressive model but a model that explains well. Last, we also note that including the lagged credit rating as a predictor of the credit rating does not seem to be common practice in the credit rating literature (see for example Hájek & Olej, 2011; Bellotti et al., 2011; Huang et al., 2004 and Yuan et al., 2018)

#### Tweets from the Companies

After collecting the financial variables and credit ratings, we check whether the companies have an official Twitter account or not and remove all the companies that do not have an official Twitter account^16^. For the companies that have a Twitter account we collect the last 3200 tweets^17^ using Twitter’s search API. We then exclude all companies that have a Twitter account but do not tweet (e.g. Apple). Given that we are able to scrape only 3200 tweets for each company at the time of data collection, for each company we also exclude all observations that relate to times before the period of the earliest tweets that we were able to capture^18^. This gives an unbalanced panel data-set^19^, where we use the first collected tweet and the last collected tweet as the cut-offs for each company’s individual time-line. Hence, only companies that have a Twitter account and tweet are considered in our analysis. Since we have an annual data-set we group these tweets into annual text variables. This means, that we aggregate each company’s tweets on an annual scale^20^.

#### Tweets about the Companies

Here, we check again whether the remaining companies have an official Twitter account or not, and remove all the companies that do not have one. In a next step, we collected all tweets that mention one of the company’s Twitter accounts using “@” (for instance “@Apple”) from 2011 until May 2019 from the University of Pennsylvania’s Twitter database^21^. We did not collect the tweets that mention one of the company’s Twitter accounts using Twitter’s official API, because Twitter’s official API did not allow to collect historical tweets using “@” at the time of data collection. We again use the date of a company’s first collected and last collected tweet as cut-offs for their individual time-lines. Hence, only companies that people tweet about are considered in our analysis. In the last step we again group the tweets into an annual variable.

### Data Preparation

It is common that Twitter users tweet irregularly. These irregularities can be arbitrary, and we also notice this pattern in our data-set for most of the companies. For example, in our data-set we have some years when there is no tweet. This means that in these years neither the companies themselves nor others who tweet about companies issued tweets, which gives a tweet frequency of zero for these years. Since we can not calculate a tweet sentiment score for non-existing tweets, and since deleting these years would have led to a huge information loss, we impute the missing sentiment scores with a value of zero.

We chose an imputation of zero and not any other imputation or interpolation method for several different reasons. First, we argue that the sentiment of a non-existent tweet is neutral and hence should be zero. Imputing or interpolating the sentiment scores with a value different to zero would require a priori knowledge on whether or not tweeting is indicative of a negative or a positive sentiment, which we do not have. Second, considering that we collected each company’s last 3200 available tweets, there may be a specific reason why a company did not tweet within a specific year which is why we should not interpolate the sentiment scores of these months or years to a value that might indicate positiveness or negativeness, while this might not necessarily be the case. For a similar reason we also imputed the years where we did not collect any tweets about a company with a value of zero. The tweets about companies were collected from a random 1% subset of all available tweets. Hence, contrary to the collection of tweets from the companies, no tweets within a year does not necessarily mean that there was no tweet, but could result from the fact that we were only able to obtain the tweets from a random 1% sample. Since we do not know what the sentiment of the missing tweets might look like and since this data restriction holds for all the tweets about companies, an imputation of zero is also sensible for this dataset.

### Data description

After the data collection and cleaning process we exclude all financial service companies^22^ from our sample, since the relationship between their ratings and the predictors is likely to differ from that for companies in other sectors (Hwang et al., 2010). We obtain two different data-sets as described in Table 2.

**Table 2:** Data overview

Twitter Data	Observations	Companies	Above AA	A	BBB	BB	Below B
From Companies	2645	699	56	358	953	866	412
About Companies	2004	420	135	400	766	473	230

Table 2 shows that the number of companies and number of observations is higher for the data-set using tweets from companies compared to the data-set using tweets about companies. We also notice that the number of observations is different within the different rating groups. Hence, we have an unbalanced dependent variable.

In addition to the information contained in Table 2 we want to descriptively analyze the differences between tweets from companies and tweets about companies in terms of their sentiment score. We do this in order to check our assumption, that tweets from companies are likely to contain a lot of advertisement and are overall more positive as opposed to tweets about companies, which we expect to be critical as well as positive towards the companies of interest. For this we calculate the average sentiment score *AS* of tweet sentiment scores *tw* per year *t* and per company *k*:

4 $$\begin{aligned} AS_{kt}=\frac{1}{f_{kt}}\sum ^{f_{kt}}_{l=1}tw_l \end{aligned}$$

where $f_{kt}$ is the number of tweets about or from company *k* in year *t* and hence $tw_l$ a single sentiment score of a tweet by/from company *k* in year *t*. Using these different annual average sentiment scores for the different companies we can calculate the overall mean, standard deviation, minimum, and maximum for tweets from and about companies and compare the results^23^. For example, the reported minimum is the minimum of the average annual sentiment score when using either tweets from or about companies. Table 3 shows the summary statistics of the general SM sentiment score and Table 4 the summary statistics of the Finance sentiment score.

Table 3 shows that the general SM sentiment score of tweets from and about companies is on average positive, where the average sentiment score using tweets about companies is slightly higher as opposed to the average sentiment score using tweets from companies. However, the standard deviation of tweets about companies is much higher as opposed to the standard deviation of tweets from companies. This shows that the average annual Twitter sentiment score has a much larger variation for tweets about companies. This higher standard deviation of tweets about companies strengthens our hypothesis that tweets about companies have a higher sentiment variability as opposed to tweets from companies, which we expect to be positive, or negative to a lesser extent, since they are issued by the companies themselves. Table 3 also confirms our assumption that tweets from companies are likely to contain advertisement, as such tweets are mostly positive or neutral as the minimum of “-1” and the maximum of “3” shows.

**Table 3:** SM sentiment score

Twitter Data	Minimum	Mean	Maximum	Standard Deviation
From Companies	-1	0.501	3	0.273
About Companies	-7.24	0.596	211	5.42

Table 4 again confirms our hypothesis that tweets from companies are mostly positive and contain advertisement. Our hypothesis implies that their average annual sentiment scores should hence have a smaller range while tweets about the companies are usually complaints or compliments about the company, its products or its services, which we expect to have a higher range. The results are confirmed by the positive average Finance sentiment score of tweets from companies, the small standard deviation and the minimum of $-1$ and maximum of 2 which are relatively close to each other. The average annual finance sentiment scores using tweets about companies have a higher range going from $-37$ to 8.75, while the mean of 0.04 is lower as opposed to using tweets from companies and the standard deviation of 1.09 much higher.

**Table 4:** Finance sentiment score

Twitter Data	Minimum	Mean	Maximum	Standard Deviation
From Companies	-1	0.157	2	0.159
About Companies	-37	0.04	8.75	1.09

### Models

**Fig. 2:**
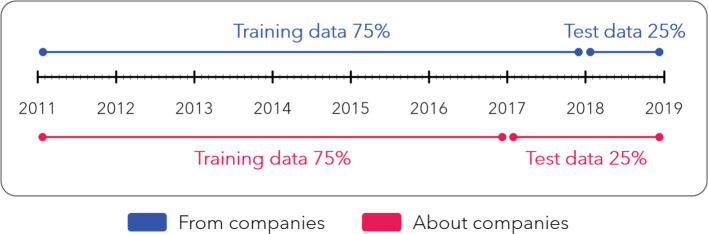
Training and test data specifications

In this section we fit the different models using a random forest classifier. We compare the predictive performance of the models with and without Twitter data using longitudinal test samples (see Jones et al., 2015) as described in Figure 2.

We split the data in 2018 for the data-set containing tweets from the companies and 2017 for the data-set containing tweets about the companies in order to obtain approximately 75% training and 25% test data.

#### Models with sentiment scores

We estimate two sets of random forest classifiers; one set where the inputs are not lagged and one set where they are lagged. In each case we estimate a base classifier that has inputs that consist of the four financial ratios and we also estimate an enhanced classifier which includes the four financial ratios plus the two twitter variables “Tweet Sentiment Score” and “Tweet Frequency”. We estimate the latter model twice, once using the sentiment score from the SM-Word-list as a predictor (SM score) and once using the sentiment score from the Finance-Word-list as a predictor (Finance score).

We first do not lag the inputs, since according to Kim and Sohn (2008) the financial variables are supposed to have an effect on the rating from the beginning of the year to the end of the year. We simply assume this holds for the Twitter variables as well. In other words, we use the explanatory variables obtained from the same year as the credit rating (*t*) to predict the credit rating (*t*). Hence, this model performs a classification rather than a prediction. We refer to this model as “Non-Lagged”.

Other authors argue that the explanatory variables should be lagged, since current values could contain information that was unknown at the time of the credit rating assignment (Bellotti et al., 2011). Since this could especially be true for the tweets we additionally fit all models using the lagged explanatory variables.

Since the random forest classifier samples different bootstrap samples each time, the model results slightly change during each run. In order to report robust results we run each random forest classifier 100 times and report the average results.

For comparing the performance of the models we use the multiclass area under the curve (AUC) (Hand & Till, 2001), the accuracy and the mean absolute error (MAE)^24^, which are all performance metrics that are typically used for ordinal responses.

To assess whether the reported differences in performance metrics are significant, we additionally run each model on 100 different bootstrap samples of the test set^25^. We choose a sample size of 400 observations without replacement for each test sample to avoid decreasing the test data size too much, but still allow for enough variation between different samples. We then test whether the difference between the models with Twitter features and the model without Twitter features is significant at 5% confidence level, using a one-sided paired t-test^26^. The asterisks in Table 5 indicate that the difference in performance metric is significant. The average results obtained from the different bootstrap test samples and the p-values of the t-tests are reported in the Appendix.

The results are reported in Table 5 and show that including information from tweets from the companies themselves and about companies improves the model performance using both sentiment scores and for non-lagged and lagged explanatory variables in terms of AUC, accuracy and MAE. Using tweets from companies, both Twitter models outperform the baseline model, where the model with the SM score performs slightly better when non-lagged predictors are used and the model with the Finance score when lagged predictors are used. Since both sentiment scores perform well this suggests that the finance word-list as well as the general word-list can successfully be used for this application. This result could also indicate that credit rating agencies include information from company’s social media accounts when performing their credit rating assessment.

Including information from tweets about companies also increases classification performance among all three performance measures for both sentiment scores when using non-lagged explanatory variables. The results when using lagged explanatory variables also show a better performance when including Twitter variables for the performance measures AUC and MAE, where the model with the Finance score slightly outperforms the model with the SM score. Only the accuracy of the baseline model is slightly higher as opposed to the models containing the Twitter variables.

**Table 5:** Model results - The models that contain Twitter variables contain the tweet frequency and one of two different tweet sentiment scores

Data	Classifier	Performance Measure	Without Twitter	With Twitter SM Score	With Twitter Finance Score
From Companies	Non-Lagged	AUC	0.794	0.813^*^	0.816^*^
		Accuracy	0.515	0.538^*^	0.531^*^
		MAE	0.595	0.556^*^	0.561^*^
	Lagged	AUC	0.830	0.835^*^	0.837^*^
		Accuracy	0.548	0.556^*^	0.554^*^
		MAE	0.568	0.534^*^	0.528^*^
About Companies	Non-Lagged	AUC	0.810	0.829^*^	0.827^*^
		Accuracy	0.532	0.546^*^	0.545^*^
		MAE	0.598	0.556^*^	0.556^*^
	Lagged	AUC	0.832	0.842^*^	0.844^*^
		Accuracy	**0.564**	0.561	0.557
		MAE	0.572	0.550^*^	0.549^*^

#### DLA - Application

In the next application, we evaluate how the performance of the models changes when including linguistic features from the tweets into the model which we obtain by applying DLA. We apply DLA to identify the linguistic features in tweets that are most predictive of yearly credit rating levels. When applying DLA we initially also included the industry division as control variable as we would expect that some linguistic features contained in the tweets could be heavily used within one industry division. For example, n-grams such as “drilling” or “mineral exploration” are more likely to occur in tweets about and from companies within the mining industry division. If many companies within this industry division would for instance have a credit rating level below B, these words would likely be identified as correlated to a credit rating level below B, even though this might not hold for all companies or companies within other industry divisions. By including the industry division as control variables we prevent this problem. However, the industry division was not found to be significant and excluding it as a control variable did not change the results. Therefore, we continue this analysis without including the industry division as a control variable. We did not include other control variables, such as the industry, which is more specific than the industry division, since including such a large number of dummy variables would not have been feasible.

Similar to the previous application, we apply DLA to lagged and non-lagged data-sets. Since we are later interested in using the identified linguistic features for out-of-time predictions, we apply DLA on a training data-set that is identical to the training data-set we will use for model estimation.

Since DLA can only be applied to binary or continuous variables, we transform the ordinal credit rating levels into binary variables as follows: “Over AA” vs. “Not Over AA”, “A” vs. “Not A”, “BBB” vs. “Not BBB”, “BB” vs. “Not BB”, “Below B” vs. “Not Below B”. We apply DLA on each of these binary variables, which results in different identified linguistic features.

The Benjamini-Hochberg (Benjamini & Hochberg, 1995) corrected significant linguistic features are displayed in word-clouds, where the color represents the p-values of the n-grams and the size represents the frequency of the n-grams in the corpus. The frequency is reported from small (infrequent) to large (frequent) and the p-values are displayed increasingly from red to blue. The word-clouds have been created using ggplot2, which uses a square root scaling by default. We further scale the font of each label so that the text area is a function of the raw size aesthetic. More information on the scaling can be found in the “ggwordcloud” package^27^. As we cannot report all significant linguistic features within one word-cloud, we limit the visualization to the 50 most frequent significant linguistic features^28^.

While analyzing the identified linguistic features within a financial context (i.e. describing which linguistic features seem sensible to be correlated to a specific credit rating level) is not a primary aim of this paper, we would expect that higher credit rating levels and credit rating upgrades are associated with words that might generally reflect good financial performance while lower credit rating levels and credit rating downgrades are associated with words that might generally reflect bad financial performance. However, since our primary aim is prediction, we will analyze the word-clouds in a general framework and propose two different strategies to select specific linguistic features that we will use as additional predictors, since including all significant linguistic features as predictors would lead to too many variables.

We use annual lagged and non-lagged tweets from the companies themselves and about the companies and show which n-grams contained in these tweets are individually most predictive of credit rating levels.

We choose only two n-grams from each resulting word-cloud to use as additional predictors. We propose to select two n-grams and not more, since this results in 10 additional predictors already, and a large number of predictors would have led to overfitting.

As we cannot select the linguistic features in terms of their marginal effects ($\beta$-coefficients of different logistic regression models cannot be compared), we propose to select the linguistic features in terms of their significance (which can be meaningfully compared across samples and models), their frequency and their generalizability across different companies and industries. We propose two different selection approaches based on these criteria and compare the results.

**In the first approach** (Approach 1) we select the two linguistic features from the 50 most frequent linguistic features of the word-clouds that have the lowest p-value and that do not relate to a specific company or industry. We have chosen these linguistic features as potential predictors for three reasons. First, we consider only linguistic features with a high frequency since these features are more representative within our sample and hence can give more reliable results as opposed to linguistic features that are used only very rarely. Second, we select features with a low p-value because this means that these linguistic features are highly significant which is a promising attribute in terms of predictive accuracy. In other words, by selecting the n-grams with the lowest p-value we can be highly confident that these have an effect on the dependent variable that is not zero. Third, we do not select features that are either directly related to a specific company or industry, or that are heavily used by only one company or one industry, since including such features in the predictive modeling framework could lead to biased results. We do this by disregarding n-grams that directly relate to a specific company or industry in the selection process. For example, we exclude n-grams such as “kindle” since this is a product of the company “Amazon” and therefore directly relates to it, and n-grams such as “vaccine” as this is a well-known product type of pharmaceutical companies and therefore directly relates to the pharmaceutical industry. Additionally, we exclude n-grams that are heavily used by only one industry or company. In other words, we exclude n-grams from the selection process that are used by one industry or company in more than 50% of observations that use this n-gram. For example, a n-gram that is mostly used by only one company over multiple years which has credit rating A could be found significant with credit rating level A, while it is in fact not the case across all companies in the sample. Similarly, this could happen with n-grams that are heavily used within one industry (note that industry is different to industry division, as it is more specific) while the companies within this industry all have the same credit rating.

**In the second approach** (Approach 2) we put more emphasis on the frequency when selecting the linguistic features. More specifically, we select the two most frequent linguistic features that are significant (Benjamini-Hochberg corrected p-value of $<0.05$) and that do not relate to a specific company or industry or that are not heavily used by only one company or one industry. We propose this second approach since the proposed linguistic features are, due to their frequency, even more representative within our sample. Furthermore, we employ this second approach as an alternative to the first approach since a very small p-value does not necessarily mean that the linguistic feature has a high effect on the dependent variable. In other words, the linguistic features selected in terms of p-value could be highly significant but could also have a small effect on the credit rating levels and therefore would not increase the predictive power a lot.

**Fig. 3:**
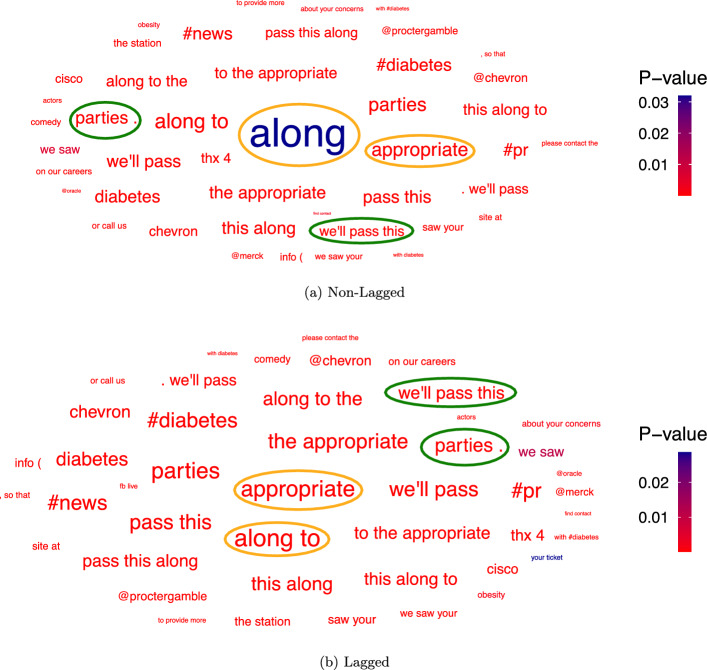
N-grams significantly correlated with Over AA - From Companies

**Fig. 4:**
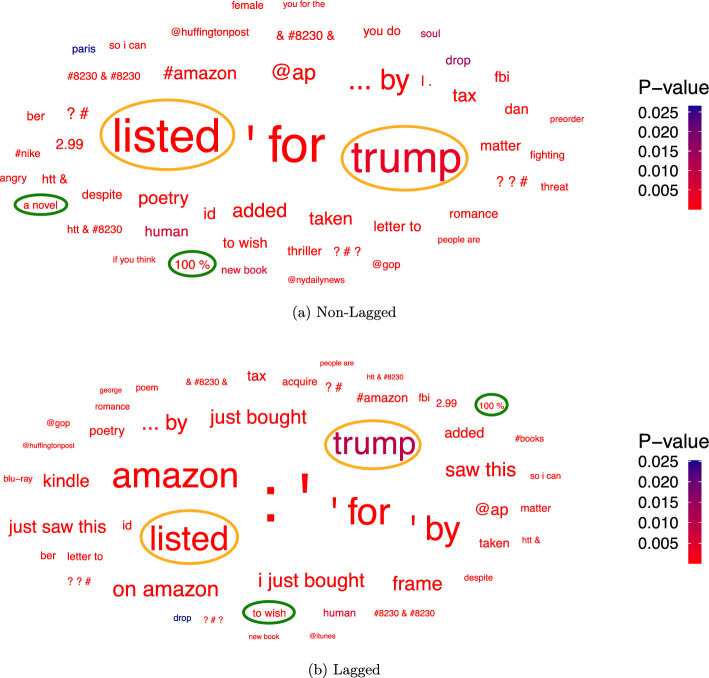
N-grams significantly correlated with Over AA - About Companies

We first apply DLA to the binary credit rating level specification “Over AA or not” and the resulting word-clouds containing the 50 most frequent significant n-grams are displayed in Figure 3 for tweets from the companies themselves and in Figure 4 for tweets about the companies. The results show that there is not a big difference between the lagged and non-lagged word-clouds as the identified linguistic features mostly coincide. This holds especially for the tweets from the companies themselves. This result is expected, as credit ratings change rarely and hence are likely to stay the same over multiple years. Additionally, the results show that some linguistic features directly refer to companies, such as “proctergamble” in Figure 3 or “amazon” in Figure 4. Also, some n-grams seem to be related to specific industries, such as the word “diabetes” in Figure 3, which we would expect to mostly appear in tweets from and about pharmaceutical (part of the Manufacturing industry division) or healthcare companies (part of the Services industry division).

As the word-clouds contain the 50 most frequent significant n-grams we can directly apply the two proposed selection approaches by selecting **1)** the two linguistic features that have the smallest p-value from each word-cloud and **2)** the two linguistic features that have the highest frequency from each word-cloud. This corresponds to selecting the two **1)** “most red” linguistic features from each word-cloud and **2)** “largest” linguistic features from each word-cloud.

The selected linguistic features of both approaches can be found in Table 6. Table 6 gives not only an overview of the selected linguistic features but also shows their frequencies and p-values. We have also marked the selected linguistic features for both approaches in the word-clouds, where the green circled n-grams have been selected according to Approach 1 and the orange circled n-grams according to Approach 2. Using the first approach we select the n-grams “we’ll pass this” and “parties .” for the non-lagged tweets from the companies themselves, as these are the n-grams with the lowest p-values that do not relate to an industry or company. The same n-grams are selected for the lagged tweets, where only the p-values slightly differ. In Figure 3 these n-grams are highlighted in green.

**Table 6:** Selected linguistic features for the dependent variable “Over AA or not”

Approach	Twitter Data	Lag	N-gram	P-Value	Frequency
Approach 1	From Companies	Non-Lagged	we’ll pass this parties .	1.962e-186 1.427e-185	596 602
		Lagged	we’ll pass this parties .	2.272e-184 1.160e-183	596 602
	About Companies	Non-Lagged	100% a novel	4.250e-96 2.502e-63	137 104
		Lagged	100% to wish	3.389e-90 2.370e-37	137 234
Approach2	From Companies	Non-Lagged	along appropriate	3.199e-02 1.127e-15	2809 1025
		Lagged	appropriate along to	1.846e-14 4.236e-18	1025 960
	About Companies	Non-Lagged	listed trump	1.571e-17 3.122e-03	1880 1821
		Lagged	listed trump	2.546e-26 9.606e-03	1880 1821

The significantly correlated n-grams of the tweets about the companies are displayed in Figure 4. We select the n-grams with the lowest p-values, which are “100%” and “a novel” from the non-lagged word-cloud and “100%” and “to wish” from the lagged word-cloud. Some n-grams have an even lower p-value, such as “ap” and “kindle”, but they directly refer to a company (The associated press) or to a product of a specific company (amazon kindle). Therefore, we did not select these n-grams.

Using the second approach we select the two most frequent significant linguistic features that do not relate to a company or industry, which are “along”/“along to” and “appropriate” from the tweets from the companies themselves and “listed” and “trump” from the tweets about the companies. We have highlighted these n-grams in Figure 3 and Figure 4 in orange. We notice that the n-grams “for” and “amazon” in Figure 4 also have a high frequency but we did not select them, since “for” is a preposition without a real meaning and “amazon” a company name.

In a next step we apply DLA to the remaining binary credit rating level specifications “A or not”, “BBB or not”, “BB or not” and “Below B or not”. The resulting word-clouds containing the 50 most frequent significant linguistic features can be found in the Appendix. Again, we select from each word-cloud the two most significant n-grams (Approach 1) and the two most frequent n-grams (Approach 2) as potential predictors. We also make sure to only select n-grams that do not relate to a specific company or industry and more information on the selection process can be found in the Appendix. Similar to Table 6, Table 8, 9, 10 and 11 in the Appendix give an overview of the selected n-grams and their frequency.

Overall, we can summarize that the selected n-grams of the lagged and non-lagged tweets are often identical, and that the frequency of n-grams from tweets from the companies themselves is overall higher as opposed to the frequency of n-grams from tweets about the companies. Both these observations are sensible, since we have more tweets from the companies themselves and since credit rating levels usually stay the same over many years. Whilst we have no evidence that the important linguistic features contained in the word-clouds change over time, because we did not consider this (and have no literature that does this either), nevertheless this is possible. We might be able to detect this by investigating word-clouds in different time-periods and by observing if the relationship between them and credit rating levels change to be found by running cross-sectional regressions for different time-periods. If we detect this, one might for example consider time-varying coefficients for the tweets in future research to account for this (as for example in Dangl & Halling 2012 and Bernoth & Erdogan 2012).

#### Models with linguistic features

As we are interested in the predictive power of the linguistic features, we fit different models using a random forest classifier and compare the performance with and without the linguistic features. We use the same model specifications as in Section 4.4.1, i.e., the same training and test data specifications, lagged and non-lagged predictors and the same baseline models. The linguistic features are included as normalized count-variables (see Section 3.3). This means that we create a variable for each selected linguistic feature by counting the occurrences of each linguistic feature over the total number of linguistic features in the tweets of a company’s year. In other words, these additional Twitter variables reflect the proportional number of times the n-grams are used by a company in the year. We initially also estimated all models by including the linguistic features without normalizing by total n-gram usage, which led to similar results.

We estimate the lagged and non-lagged model for both selections of linguistic features: the linguistic features that have been selected according to Approach 1 and the linguistic features which we selected according to Approach 2. We estimate each model using a random forest classifier and again we run the random forest classifier 100 times and report the average results, since these are more robust compared to the results of a single run. We compare the performance of the models in terms of multiclass AUC, accuracy and MAE, since we determine the success of a model in terms of predictive accuracy rather than test hypotheses. Similarly to Table 5, the asterisk indicates whether or not the difference in results is statistically significant at 5% confidence level.

The model results are displayed in Table 7. We find, that the models containing the selected linguistic features outperform the baseline models in terms of AUC, accuracy and MAE in all cases when using linguistic features from tweets from the companies themselves and in almost all cases when using linguistic features from tweets about the companies. The best results are achieved when including the linguistic features that have been selected in terms of frequency (Approach 2), as including these leads to an increase in AUC and accuracy and a decrease in MAE across all model specifications. We conclude that overall the selection in terms of frequency (Approach 2) is more appropriate as opposed to the selection in terms of p-value (Approach 1). This is sensible since significant n-grams with a higher frequency are more representative in the corpus and hence more likely to have an effect.

**Table 7:** Model results - The models that contain Twitter variables contain the linguistic features selected according to Approach 1 or Approach 2

Data	Classifier	Performance Measure	Without Twitter	Selected N-grams Approach 1	Selected N-grams Approach 2
From Companies	Non-Lagged	AUC	0.794	0.810^*^	0.838^*^
		Accuracy	0.515	0.518^*^	0.577^*^
		MAE	0.595	0.591^*^	0.494^*^
	Lagged	AUC	0.830	0.842^*^	0.854^*^
		Accuracy	0.548	0.553^*^	0.566^*^
		MAE	0.568	0.548^*^	0.513^*^
About Companies	Non-Lagged	AUC	0.810	0.815^*^	0.819^*^
		Accuracy	0.532	0.530	0.536^*^
		MAE	0.598	0.597	0.583^*^
	Lagged	AUC	0.832	0.837^*^	0.845^*^
		Accuracy	**0.564**	0.551	**0.564**
		MAE	0.572	0.587	0.568^*^

Comparing these results with the sentiment score models from Section 4.4.1 (see Table 5) shows that the models containing the linguistic features from tweets from the companies themselves perform better as opposed to the models containing the sentiment scores from tweets from the companies themselves. This result confirms the assumption that specific linguistic features are more appropriate for prediction as opposed to an overall sentiment score since tweets from companies are issued by the companies themselves which makes them to some extend biased. Also, this result confirms our overall assumption that information from tweets from companies are predictive of credit ratings, since tweets from the companies themselves reflect important aspects of the companies that could be related to their performance and therefore also to their credit ratings, such as their marketing strategy.

When comparing the results of the models using tweets about the companies with Table 5 we notice that even though the models containing the linguistic features outperform the baseline models, in most cases they do not outperform the models containing the different sentiment scores. Tweets about companies are often complaints or praises about their services or products, which therefore reflect the companies popularity which relates to their financial performance and hence their credit rating. It makes sense that the models containing the sentiment scores outperform the models containing the linguistic features, since an overall sentiment score is calculated considering all words in a tweet, which is why it can better capture a tweet’s message.

Overall, we summarize that linguistic features from tweets from and about companies are predictive of credit rating levels, since models that contain these features outperform baseline models that omit them. In most cases, the models containing the linguistic features also outperform other models containing information from Twitter, such as the tweet frequency or a tweet sentiment score.

While the observed improvements in model performance from incorporating Twitter-based features may appear small in absolute terms, their implications can be financially significant in applied settings. As formalized in the cost-sensitive loss function presented in Appendix 5.2, even small gains in classification accuracy can lead to a substantial reduction in expected economic losses when large portfolio exposures are involved. Although credit rating changes are relatively infrequent, their impact can be significant. Avoiding even a small number of costly misclassifications annually can justify the data acquisition and processing costs associated with incorporating Twitter-based features.

The results overall confirm the findings of Yuan et al. (2018) that information from Twitter can increase the predictive accuracy of corporate credit rating levels. However, the improvements in accuracy by Yuan et al. (2018) are larger than the improvements that we achieve when using a random forest classifier and information contained in tweets about companies as predictors. For example, the highest improvement in accuracy we achieve for this model specification is 2.63% when including non-lagged Twitter variables, while Yuan et al. (2018) achieved an improvement in accuracy of 4.02%. These improvement differences could result from using different Twitter variables, annual data as opposed to quarterly data and only having a random sample of 1% of tweets.

We extend the results by Yuan et al. (2018) by showing that not only information contained in tweets about companies is predictive of credit ratings, but also information contained in tweets from the companies themselves. For example, we show that using information contained in tweets from the companies themselves as additional predictors can achieve an improvement in accuracy that is close to Yuan et al. (2018) (4.46% for non-lagged predictors). Additionally, we extend the study by Yuan et al. (2018) by showing that other information from Twitter, such as simple dictionary based sentiment scores and the tweet frequency, can improve the prediction of corporate credit ratings. Last, we also show that the Twitter variables can improve the prediction of credit ratings not only in terms of accuracy, but also in terms of AUC and MAE and we show that the improvements overall hold for non-lagged and lagged predictors. We also extend the study by Yuan et al. (2018) by showing that linguistic features in tweets can further improve the prediction of corporate credit ratings.

## Conclusion

This paper shows that novel information from Twitter can improve the prediction/classification of a company’s credit rating level and that different sources of tweets and information contained in the tweets affect predictive accuracy to different extents. We estimate random forest classifier models and we include as inputs explanatory variables of the same year as the dependent variable (non-lagged) and explanatory variables from the year before the dependent variable (lagged). We estimate a baseline model and compare it to the performance of models that additionally contain different Twitter sentiment scores and the tweet frequency or linguistic features contained in the tweets. Additionally, we differentiate between tweets from companies, which are a part of a company’s marketing strategy and tweets about companies, which convey external evaluations by the public. We can draw five main conclusions.

First, we conclude that information contained in tweets from and about companies are predictive of corporate credit rating levels, as the models that contain information from Twitter have an overall higher AUC, Accuracy and MAE compared to models that omit these features. This holds for models where we used non-lagged explanatory variables and for models where we used lagged explanatory variables. Although the differences in performance between models incorporating Twitter data and those excluding it are relatively small, they are statistically significant. The findings suggest that tweets contain valuable predictive information about corporate credit ratings and consequently, firm risk, which aligns with the wisdom of crowds theory (Lerner & Tirole, 2002) when considering information from tweets about companies. When considering tweets from the companies themselves, this phenomenon can be attributed to firms deliberately signaling their quality via Twitter, as also shown in the work by Jung et al. (2018). Since both sources of tweets are publicly available, they could both be considered in the rating assessment of credit rating agencies which could also explain the predictive power.

Second, when focusing on the Twitter sentiment scores as predictors, we conclude that even though the word-list choice gives slightly different results for different sources of tweets (e.g. from the companies themselves or about the companies), both choices of word-lists perform equally well. This result is sensible, since tweets are usually written in a more general language, which explains why the SM word-list performs well, but since the predictions are performed in a financial context it makes sense that the Finance wordlist also performs well.

Third, we conclude that when using linguistic features from Twitter as additional predictors for corporate credit rating levels, selecting the features in terms of frequency, leads to better results than selecting the features in terms of p-value only. As linguistic features with a high frequency are more representative in the corpus, it makes sense that these linguistic features have an overall greater effect.

Fourth, we conclude that the information contained in the tweets (i.e. sentiment scores or linguistic features) that best predicts credit rating levels should be chosen according to the source of the tweets (i.e. tweets from companies or about companies). For example, the results show that the models containing linguistic features of tweets from the companies themselves outperform the models containing the sentiment scores and the tweet frequency. This can be explained by the fact that tweets from companies are largely positive and, therefore, a sentiment score based on positive and negative words might not be as informative as specific selected keywords. However, in most cases, the models containing the sentiment scores and the tweet frequency using tweets about companies outperform those that contain linguistic features from tweets about companies. This could be explained by the fact that tweets about companies are issued by a large number of different people, and hence differ in terms of language. Therefore, a sentiment score that roughly indicates whether a tweet is overall positive or negative and the tweet frequency are slightly better indicators of credit rating levels than specific linguistic features contained in these tweets are.

Fifth, we conclude that the performance between models containing lagged and non-lagged predictors in terms of AUC, accuracy and MAE is very similar. We believe this result is primarily driven by the relatively infrequent changes in credit ratings, especially since we are using panel data. Hence, the lagged and non-lagged predictors are likely to predict the same credit rating level and therefore should perform equally.

Different stakeholders in the credit rating industry could make use of the results that we have obtained. While the incremental improvement in model accuracy from the new features is small, the increase in predictive accuracy is statistically significant. Therefore, the additional data could lead to a more robust and reliable credit rating prediction model that credit rating agencies could use when developing a credit rating classification.

Investors could use our results to get an idea of what a company’s future credit rating level might look like and make investment decisions based on this, as even a small improvement in accuracy could significantly reduce the risk of major investment losses due to misjudging creditworthiness. For instance, a 2% improvement in prediction accuracy could prevent significant losses in high-risk sectors, where the cost of misclassifying a company’s creditworthiness is much higher, especially when making large investments in extensive portfolios. However, since Twitter data needs to be acquired, the benefits of adding these features should be weighed against their cost. This evaluation is unique to each investor’s portfolio and strategy, making it difficult to generalize; nevertheless, a cost-benefit analysis and sensitivity or scenario analysis are essential to quantify the financial impact of improved accuracy and compare it to the cost of acquiring the data.

In addition to the credit rating industry and investors, the companies themselves could be interested in our findings, since they could adjust their social media behavior in order to receive a higher credit rating level. However, our results could also be of interest to other researchers that use Twitter for financial predictions, as we show that not only standard methods, such as sentiment analysis, can be successfully used to create predictors within a financial context, but also other methods, such as DLA. Researchers could explore whether DLA can be applied to tweets for the prediction of other dependent variables, such as stock price changes or bankruptcy.

While this study provides insights into the predictive ability of Twitter data for corporate credit ratings, the feature space used can be further extended by incorporating LLMs, which could provide a more comprehensive understanding. Specifically, LLMs may be leveraged in future research to generate credit rating specific intents from the tweets. These intents can then be used as additional predictors in the model. Finally, it would be interesting to investigate whether fine-tuning an LLM using the credit rating level as the target output could provide even better results.

## Figures and Tables

**Fig. 5:**
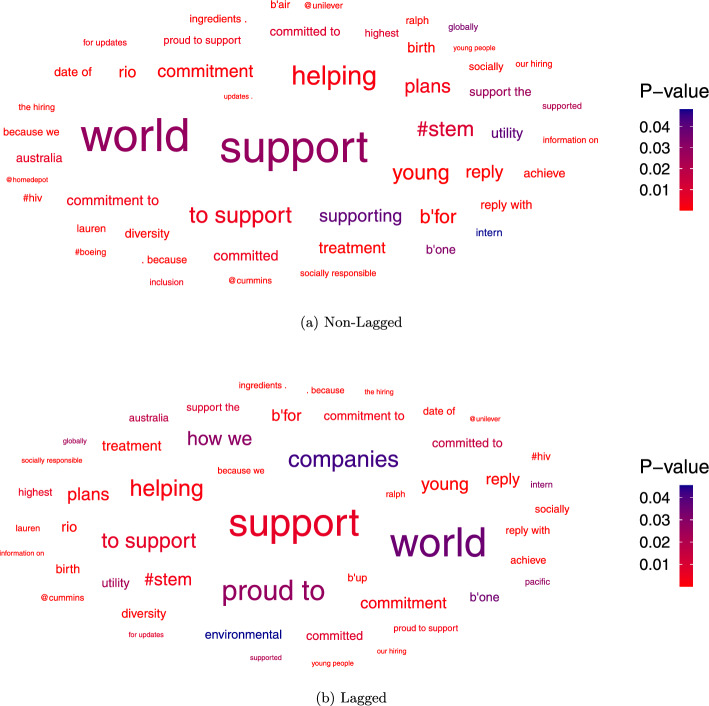
N-grams significantly correlated with A - From Companies

**Fig. 6:**
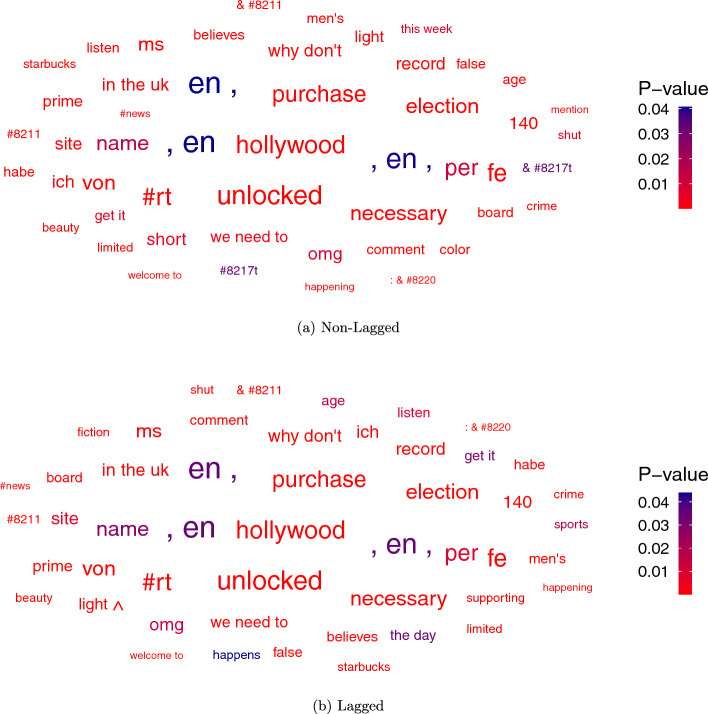
N-grams significantly correlated with A - About Companies

**Fig. 7:**
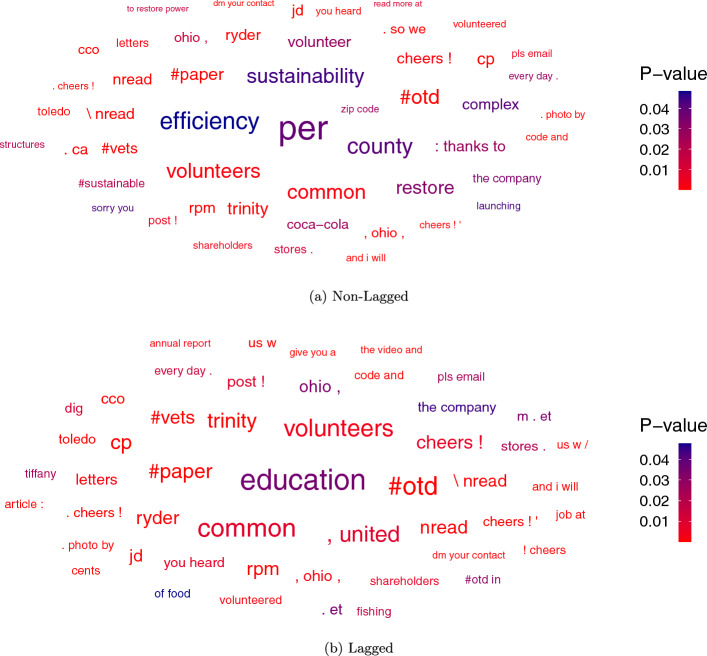
N-grams significantly correlated with BBB - From Companies

**Fig. 8:**
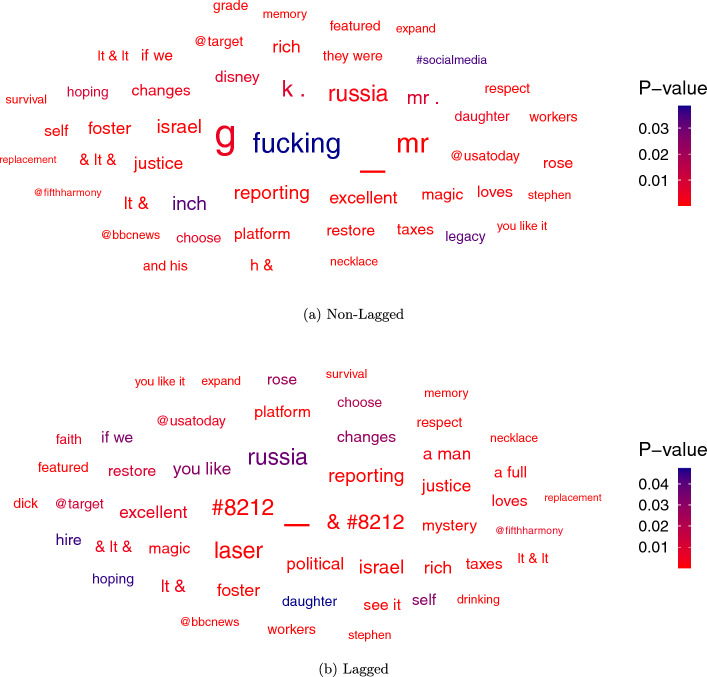
N-grams significantly correlated with BBB - About Companies

**Fig. 9:**
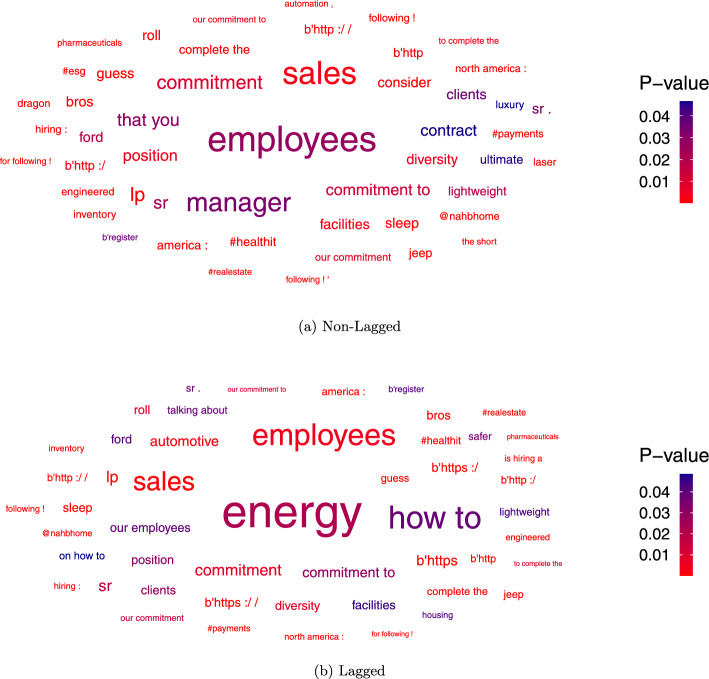
N-grams significantly correlated with BB - From Companies

**Fig. 10:**
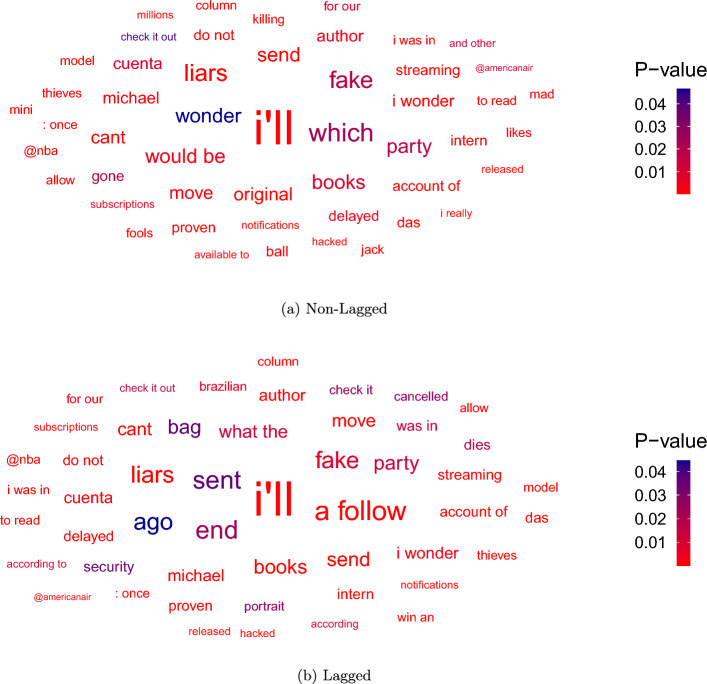
N-grams significantly correlated with BB - About Companies

**Fig. 11:**
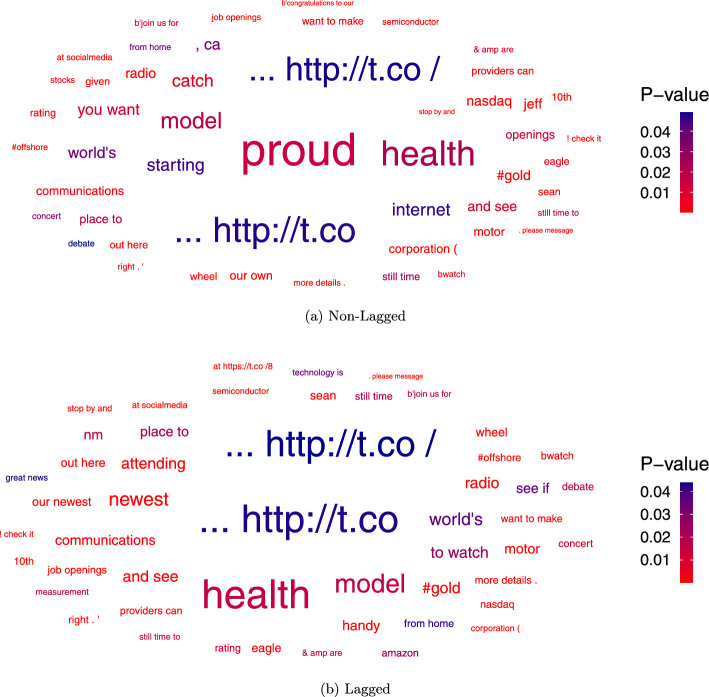
N-grams significantly correlated with Below B - From Companies

**Fig. 12:**
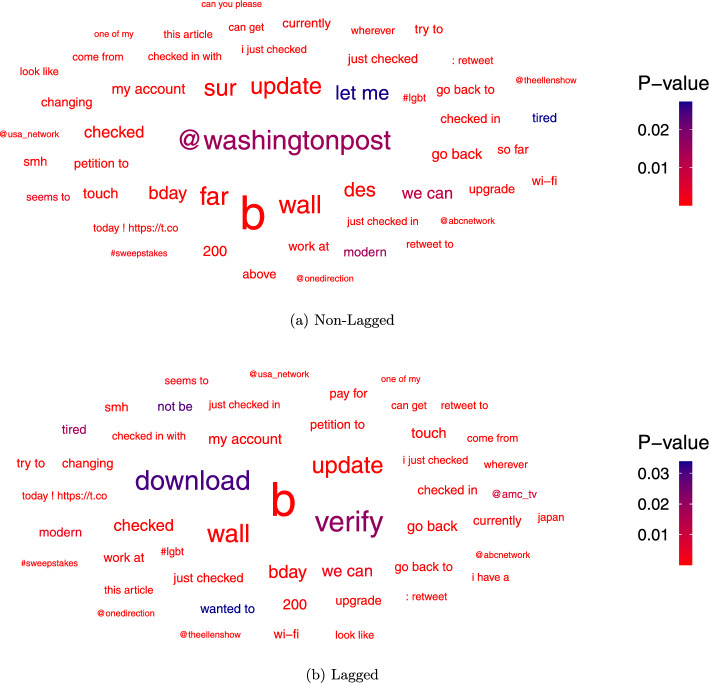
N-grams significantly correlated with Below B - About Companies

## Data Availability

We cannot directly share the data, as the financial data was acquired from Refinitiv and the Twitter data was obtained via Twitter’s official API and from the University of Pennsylvania’s Twitter database. When collecting the data via Twitter there are downloading restrictions when using the free API which is why the same data cannot be acquired without a subscription (https://developer.twitter.com/en/products/twitter-api). To recreate the dataset that was used for this study, we suggest following the data collection process as described in Section 4.1. The exact variable names of the financial variables from Refinitiv are WC03151 (Working Capital), WC03495 (Retained Earnings), WC02999 (Total Assets), WC18191 (Earnings before interest and taxes), WC03351 (Total liabilities) and Market value.

## Appendix

### Word-clouds of the remaining credit rating level specifications

#### A

The significant linguistic features ($p<0.05$, Bonferroni p-corrected) for the binary dependent variable “Credit Rating A or not” are displayed in Figure 5 and in Figure 6. The results in Figure 5 and Figure 6 show again that the non-lagged and lagged word-clouds are nearly identical. In Figure 5 we notice that some of the identified linguistic features directly relate to companies (e.g. homedepot, unilever), which we will not consider when selecting appropriate n-grams as predictors. The linguistic features we select in terms of frequency and correlation strength (Approach 1) are “ingredients .” and “our hiring” for both lagged and non-lagged data using tweets from the companies themselves. The linguistic features we select in terms of frequency (Approach 2) are “support” and “world” for both lagged and non-lagged data. A summary of the selected linguistic features is displayed in Table 8.

Using tweets about companies we select the n-grams “unlocked” and “record” for the lagged and non-lagged tweets when considering the p-value and the frequency (Approach 1) and “unlocked” and “purchase” when only considering the frequency (Approach 2), which are also displayed in Table 8. We did not select “hollywood” even though it has a high frequency, as this word relates to a specific industry. Other n-grams with lower p-values have also not been selected, as they directly relate to a company (starbucks) or are not part of the English language (ich, habe).

**Table 8:** Selected linguistic features for the dependent variable “A or not”

Approach	Twitter Data	Lag	N-gram	P-Value	Frequency
Approach 1	From Companies	Non-Lagged	ingredients . our hiring	1.121e-106 1.045e-78	772 501
		Lagged	ingredients. our hiring	1.545e-95 9.829e-80	772 501
	About Companies	Non-Lagged	unlocked record	1.206e-45 1.917e-36	881 347
		Lagged	unlocked record	3.862e-41 5.125e-37	881 347
Approach 2	From Companies	Non-Lagged	support world	2.835e-02 2.835e-02	12194 10122
		Lagged	support world	6.284e-03 3.493e-02	12189 10119
	About Companies	Non-Lagged	unlocked purchase	1.206e-45 7.885e-06	881 752
		Lagged	unlockedpurchase	3.862e-41 8.198e-07	881 752

#### BBB

The linguistic features that are individually most correlated with credit rating level BBB are displayed in Figure 7 and Figure 8 for the different sources of tweets. The largest number of companies has credit rating level BBB which is why the dependent variable “BBB or not” is more balanced as opposed to the other binary dependent variables.

We notice that the word-clouds between the lagged and non-lagged tweets are again very similar, but not as similar as in the previous word-clouds.

From Figure 7 we select the n-grams “code and” and “volunteered” from the non-lagged word-cloud when considering the frequency and the p-value (Approach 1), as the n-grams with lower p-values are either relating to companies (toledo), industries (rpm) or are simply not sensible (nread). For similar reasons we select the n-grams “article:” and “job at” from the lagged word-cloud.

When considering only the frequency (Approach 2) we select “efficiency” and “sustainability” from the non-lagged word-cloud and “volunteers” and “education” from the lagged word-cloud.

From Figure 8 containing significantly correlated n-grams from tweets about companies we select the most significant n-grams “survival” and “you like it” from the non-lagged and the lagged word-cloud. In terms of frequency we select “fucking” and “reporting” from the non-lagged word-cloud and “reporting” and “you like” from the lagged word-cloud. A summary of the selected linguistic features is displayed in Table 9.

**Table 9:** Selected linguistic features for the dependent variable “BBB or not”

Approach	Twitter Data	Lag	N-gram	P-Value	Frequency
Approach 1	From Companies	Non-Lagged	code and volunteered	9.077e-07 9.866e-04	392 367
		Lagged	article : job at	2.540e-08 4.898e-07	307 300
	About Companies	Non-Lagged	survival you like it	1.173e-14 2.465e-14	91 90
		Lagged	survival you like it	8.921e-11 2.321e-10	91 90
Approach 2	From Companies	Non-Lagged	efficiency sustainability	4.844e-02 4.423e-02	2085 2000
		Lagged	education volunteers	3.509e-02 3.453e-03	2252 1572
	About Companies	Non-Lagged	fuckingreporting	3.861e-02 8.724e-09	439 263
		Lagged	reportingyou like	4.756e-06 3.037e-02	263 194

#### BB

The results of DLA applied to the credit rating level “BB or not” are displayed in Figure 9 for the linguistic features from the data-set using tweets from the companies themselves and in Figure 10 for the linguistic features from the data-set using tweets about the companies.

Similar to the other obtained word-clouds, there are n-grams contained in Figure 9 and 10 that directly relate to a company (e.g. americanair) or industry (e.g. pharmaceuticals). We will disregard these n-grams in order to avoid a bias in our results. Additionally, we notice again a high similarity between the lagged and non-lagged word-clouds, especially when tweets about the companies are used.

The linguistic features we select in terms of p-value and frequency are displayed in Table 10.

For the non-lagged tweets from the companies themselves the linguistic features “automation ,” and “following !” have been selected in terms of p-value and frequency, and “employees” and “sales” in terms of frequency. For the lagged tweets from the companies themselves we select “#payments” and “hiring:” and “employees” and “sales” respectively.

The linguistic features we select from Figure 10 are the following: We select “fools” and “liars” from the non-lagged word-cloud and “liars” and “notifications” from the lagged word-cloud when using Approach 1 for selection. Using Approach 2 we select “fake” and “wonder” from the non-lagged word-cloud and “a follow” and “end” from the lagged word-cloud.

#### Below B

The word-clouds that we have obtained when correlating the linguistic features in tweets to the credit rating level “Below B” are displayed in Figures 11 and 12. The credit rating group “Below B” is our lowest credit rating group which relates to the most risky companies in our sample. Again, the word-clouds of the lagged and non-lagged tweets are very similar, which holds especially for the tweets from the companies themselves.

Figure 11 contains the 50 most frequent significantly correlated n-grams that result from applying DLA to the tweets from the companies themselves. The n-grams with the lowest p-values that do not relate to a specific industry or company are “corporation (” and “nasdaq” for the non-lagged word-cloud and “corporation (” and “providers can” for the lagged word-cloud. We do not select features such as “...http://t.co” as they do not have a real meaning. Considering only the frequency, we select “proud" and “health", and “health" and “model" for the non-lagged and lagged tweets respectively. We further notice the n-gram “proud” which is displayed in the middle of the non-lagged word-cloud. Additional analyses showed, that the n-gram “proud”, which is displayed in the middle of the non-lagged word-cloud, has a negative $\beta$-coefficient. This is sensible, as we would assume that words that are generally perceived as positive are more likely to be correlated with less risky companies as opposed to high risk companies.

**Table 10:** Selected linguistic features for the dependent variables “BB or not”

Approach	Twitter Data	Lag	N-gram	P-Value	Frequency
Approach 1	From Companies	Non-Lagged	following ! ’ automation,	5.455e-40 1.162e-34	426 441
		Lagged	#payments hiring:	1.011e-21 1.178e-11	645 515
	About Companies	Non-Lagged	foolsliars	7.842e-53 3.901e-47	138 401
		Lagged	liarsnotifications	2.457e-48 4.602e-43	401 182
Approach 2	From Companies	Non-Lagged	employeessales	2.699e-02 2.573e-03	7107 3768
		Lagged	employees sales	4.744e-03 7.654e-04	7100 3760
	About Companies	Non-Lagged	wonderfake	4.675e-02 1.847e-02	448 410
		Lagged	a followend	6.495e-09 2.360e-02	762 479

An overview of the selected linguistic features, their p-values and frequencies is displayed in Table 11.

From Figure 12 we select the n-grams “checked in with” and “work at” from the non-lagged and lagged word-cloud, as these n-grams have the lowest p-value (Approach 1). We did not select n-grams with low p-values that are very similar to “checked in with”, such as “i just checked” as these could exist jointly, as for example in “i just checked in with”. Using the second approach we select “my account” and “update” from the non-lagged word-cloud and “download” and “verify” from the lagged word-cloud (see Table 11).

### Test set bootstrap sample results

Table 12 and Table 13 present the average results from the 100 bootstrap samples of the test set. The tables also include the p-values from the one-sided t-tests applied to the results. These t-tests compare the performance of the models with Twitter features against the model without Twitter features and the null hypothesis assumes that the models with Twitter features perform equal to or worse than the model without Twitter features^29^. Overall, the results in Table 12 and Table 13 align with the results derived from the full test set in Table 5 and Table 7. While the exact numbers may vary due to different test sets, the overall pattern and size of the differences between models are comparable.

**Table 11:** Selected linguistic features for the dependent variables “Below B or not”

Approach	Twitter Data	Lag	N-gram	P-Value	Frequency
Approach 1	From Companies	Non-Lagged	corporation ( nasdaq	5.411e-148 1.940e-59	1030 1010
		Lagged	corporation ( providers can	4.951e-126 1.772e-43	381 689
	About Companies	Non-Lagged	checked in with work at	1.125e-93 1.056e-60	157 150
		Lagged	checked in with work at	2.523e-91 4.166e-60	157 150
Approach 2	From Companies	Non-Lagged	proudhealth	1.517e-02 2.319e-02	8947 5376
		Lagged	health model	1.440e-02 1.579e-02	5375 2355
	About Companies	Non-Lagged	updatemy account	1.022e-14 1.981e-34	463 251
		Lagged	downloadverify	3.043e-02 2.033e-02	709 620

**Table 12:** Average bootstrap results - The models that contain Twitter variables contain the tweet frequency and one of two different tweet sentiment scores

Data	Classifier	Performance Measure	Without Twitter	With Twitter SM Score	With Twitter Finance Score
From Companies	Non-Lagged	AUC	0.793	0.809 P < 2.2e-16	0.813 P < 2.2e-16
		Accuracy	0.521	0.547 P < 2.2e-16	0.531 P = 5.9e-14
		MAE	0.587	0.538 P < 2.2e-16	0.565 P < 2.2e-16
	Lagged	AUC	0.829	0.835 P < 2.2e-16	0.836 P < 2.2e-16
		Accuracy	0.544	0.556P < 3.454e-15	0.552 P = 1.909e-08
		MAE	0.577	0.526 P < 2.2e-16	0.534P < 2.2e-16
About Companies	Non-Lagged	AUC	0.813	0.826P < 2.2e-16	0.831 P < 2.2e-16
		Accuracy	0.540	0.550P = 1.054e-15	0.547P = 7.036e-09
		MAE	0.590	0.556P < 2.2e-16	0.555P < 2.2e-16
	Lagged	AUC	0.833	0.846 P < 2.2e-16	0.841P < 2.2e-16
		Accuracy	0.569	0.563P = 1	0.552P = 1
		MAE	0.569	0.567 P < 0.05045	0.541P < 2.2e-16

**Table 13:** Average bootstrap results - The models that contain Twitter variables contain the linguistic features selected according to Approach 1 or Approach 2

Data	Classifier	Performance Measure	Without Twitter	Selected N-grams Approach 1	Selected N-grams Approach 2
From Companies	Non-Lagged	AUC	0.793	0.828 P < 2.2e-16	0.840 P < 2.2e-16
		Accuracy	0.521	0.557 P < 2.2e-16	0.579P < 2.2e-16
		MAE	0.587	0.542 P < 2.2e-16	0.503 P < 2.2e-16
	Lagged	AUC	0.829	0.835 P < 2.2e-16	0.836 P < 2.2e-16
		Accuracy	0.544	0.567P < 2.2e-16	0.581 P < 2.2e-16
		MAE	0.577	0.526 P < 2.2e-16	0.534 P < 2.2e-16
About Companies	Non-Lagged	AUC	0.813	0.815P = 4.416e-14	0.821 P < 2.2e-16
		Accuracy	0.540	0.529P = 1	0.545P = 2.155e-09
		MAE	0.590	0.599 P = 1	0.568P < 2.2e-16
	Lagged	AUC	0.833	0.838P = 4.416e-14	0.843 P < 2.2e-16
		Accuracy	0.569	0.565 P = 1	0.570P = 0.02856
		MAE	0.569	0.565 P = 2.686e-07	0.548 P < 2.2e-16

### Cost-Sensitive Loss Function for Credit Rating Prediction

To assess the practical relevance of Twitter-based features, we define a cost-sensitive loss function that reflects the economic consequences of misclassifying credit ratings. Let:

- $$y_i \in \mathcal {R}$$ be the true credit rating of firm *i*, where $$\mathcal {R} = \{\text {Above AA}, \text {A}, \dots , \text {Below B}\}$$,
- $$\hat{y}_i$$ be the predicted rating for company *i*,
- $$p_i$$ be the dollar exposure to company *i* (e.g., portfolio weight),
- *r*(*y*) maps rating *y* to an ordinal scale (e.g., Above AA = 1, ..., Below B = 5),
- $$\alpha $$ be a scaling factor (e.g., cost of one-notch error per $1 of exposure),
- $$\lambda $$ be the fixed cost of acquiring Twitter data.

We define the misclassification cost as:

$$ C(y_i, \hat{y}_i) = {\left\{ \begin{array}{ll} 0 & \text {if } y_i = \hat{y}_i \\ \alpha \cdot |r(y_i) - r(\hat{y}_i)| & \text {otherwise} \end{array}\right. } $$

The total expected economic loss for a model is then:

$$ \mathcal {L} = \sum _{i=1}^{N} C(y_i, \hat{y}_i) \cdot p_i + \lambda $$

We define two losses:

$$\begin{aligned} \mathcal {L}_{\text {baseline}}&= \sum _{i=1}^{N} C(y_i, \hat{y}_i^{\text {base}}) \cdot p_i \\ \mathcal {L}_{\text {Twitter}}&= \sum _{i=1}^{N} C(y_i, \hat{y}_i^{\text {twitter}}) \cdot p_i + \lambda \end{aligned}$$

The net benefit of using Twitter data is:

$$ \Delta \mathcal {L} = \mathcal {L}_{\text {base}} - \mathcal {L}_{\text {twitter}} $$

If $\Delta \mathcal {L} > 0$, the use of Twitter features leads to a reduction in expected economic loss despite their cost.

**Illustrative Example.** To demonstrate the economic relevance of the observed accuracy improvement, we consider a realistic credit portfolio of $1 billion distributed across 1,000 firms, implying an exposure of $1,000,000 per firm.

Including Twitter-based features increases the model’s accuracy from 0.515 to 0.538 for the non-lagged model using tweets from the companies themselves and the Twitter SM score (see Table 5). This is an improvement of 2.3 percentage points. Applied to 1,000 firms, this corresponds to approximately 23 additional correct rating predictions.

To translate this into economic terms, we interpret these as avoided misclassifications. Under a conservative assumption in which all corrected errors correspond to one-notch differences, the associated savings are:

$$ 23 \cdot (0.01 \cdot 1{,}000{,}000) = 230{,}000. $$

Subtracting an annual Twitter data cost of $100,000 yields a net benefit of approximately $130,000.

This example illustrates that even modest statistical improvements in rating accuracy can generate economically meaningful gains when applied to large credit portfolios. In addition to making the predicted value of a portfolio more accurate and thus revealing opportunities for profitable arbitrage and trading of the bond portfolios themselves, more accurate valuation, of the magnitude we have exemplified, may affect the valuation of the underlying company whose bonds are being rated. These magnitudes could influence the likelihood of the company being acquired, its ability to issue more bonds or obtain other types of financing, and the willingness of suppliers to sign supply contracts.

## Footnotes

1 https://www.statista.com/statistics/303681/twitter-users-worldwide/; accessed 07.06.2023.

2 https://cran.r-project.org/web/packages/randomForest/randomForest.pdf.

3 Website: https://www.cs.uic.edu/~liub/FBS/sentiment-analysis.html.

4 All our analyses been performed using functions from DLATK https://dlatk.wwbp.org/index.html.

5 http://sentiment.christopherpotts.net/code-data/happyfuntokenizing.py.

6 For example, in our study we explore around 13,000 features contained in tweets about companies and around 60,000 features contained in tweets from companies.

7 This is done by adjusting the color intensity (frequency) and the size of each significant language feature (correlation strength/ effect).

8 These statistical algorithms are used if control variables need to be specified.

9 This setting cannot be changed.

10 The NYSE is the biggest stock exchange by market capitalization of listed companies in the world and lists over 3,000 companies, containing some of the biggest US companies.

11 The NASDAQ is the second biggest stock exchange in the world, listing around 2,700 companies, ranging from small-cap to large-cap stocks.

12 Application-Programming-Interface https://developer.Twitter.com/en/docs/tweets/search/overview.

13 See https://www.standardandpoors.com/en_US/web/guest/article/-/view/sourceId/504352.

14 https://www.refinitiv.com/en/financial-data/company-data/fundamentals-data/worldscope-fundamentals.

15 Similar to the study by Kim and Sohn (2008).

16 We do this check by scraping Google search with the query “**Company** Twitter Account US”, where “**Company**” is the respective company name. Since the first search result is almost always the Twitter account if it exists, and since the URL of that is always https://Twitter.com/company?lang=en where company is the respective company name, we simply extract the URL of the first search result for each query. Then, we delete https://Twitter.com/ and url?lang=en in order to get the official Twitter account name for each company.

17 This is the limit set by Twitter at the time of data collection.

18 For example, we collect credit rating data and the financial ratios for Company A from 2010 until 2019. However, due to the 3200 tweet restriction we could not collect tweets that go further back than 2016. Hence, we remove all the observations before 2016.

19 Other examples why the data-set is unbalanced: one company tweeted irregularly from 2011 until 2019. For this company we have observations over 9 years, even though there are many days and even months when the company did not tweet at all. Another company only tweeted a few times in 2017, hence we have only observations for 2017. Another company tweets a lot and hence the last 3200 tweets we were able to collect only went back until 2018.

20 For example, all tweets from Company A in 2016 are aggregated into one text for the annual data-set.

21 A database where 1% of all tweets are randomly collected by month.

22 Companies with SIC code between 6000-6799.

23 Since we used the average values for a single tweet, observations for which we did not obtain any tweets are disregarded in this calculation.

24 Assuming an equal distance between classes.

25 For computational reasons, we train each model in this experiment with a fixed seed and disregard the previously described changes that occur when using a random forest classifier.

26 We use a paired test because for each observation we have a prediction from the model without Twitter features and from the model with Twitter features.

27 https://cran.r-project.org/web/packages/ggwordcloud/vignettes/ggwordcloud.html.

28 We focus on the 50 most frequent significant n-grams, since n-grams with a high frequency are expected to be more meaningful.

## References

[CR1] Allison, P. D. (1999). Comparing logit and probit coefficients across groups. *Sociological Methods and Research,**28*(2), 186–208.

[CR2] Almamy, J., Aston, J., & Ngwa, L. N. (2016). An evaluation of altman’s z-score using cash flow ratio to predict corporate failure amid the recent financial crisis: Evidence from the uk. *Journal of Corporate Finance,**36*, 278–285.

[CR3] Altman, E. I. (1968). Financial ratios, discriminant analysis and the prediction of corporate bankruptcy. *The Journal of Finance,**23*(4), 589–609.

[CR4] Altman, E. I., Danovi, A., & Falini, A. (2013). *Z-score models’ application to italian companies subject to extraordinary administration*. Corporate Finance: Capital Structure & Payout Policies eJournal.

[CR5] Altman, E. I., & Rijken, H. A. (2004). How rating agencies achieve rating stability. *Journal of Banking & Finance,**28*(11), 2679–2714. Recent Research on Credit Ratings.

[CR6] Amato, J., & Furfine, C. (2004). Are credit ratings procyclical? *Journal of Banking & Finance,**28*(11), 2641–2677.

[CR7] Beaver, W. H. (1966). Financial ratios as predictors of failure. *Journal of Accounting Research,**4*, 71–111.

[CR8] Bellotti, T., Matousek, R., & Stewart, C. (2011). A note comparing support vector machines and ordered choice models predictions of international banks ratings. *Decision Support Systems,**51*(3), 682–687.

[CR9] Benjamini, Y., & Hochberg, Y. (1995). Controlling the false discovery rate: A practical and powerful approach to multiple testing. *Journal of the Royal Statistical Society: Series B (Methodological),**57*(1), 289–300.

[CR10] Bernoth, K., & Erdogan, B. (2012). Sovereign bond yield spreads: A time-varying coefficient approach. *Journal of International Money and Finance,**31*(3), 639–656. Financial Stress in the Eurozone.

[CR11] Bharath, S.T., & Shumway, T. (2004). Forecasting default with the kmv-merton model. *AFA 2006 Boston Meetings Paper*.

[CR12] Blume, M. E., Lim, F., & Mackinlay, A. C. (1998). The declining credit quality of u.s. corporate debt: Myth or reality? *The Journal of Finance,**53*(4), 1389–1413.

[CR13] Bollen, J., Mao, H., & Zeng, X. (2011). Twitter mood predicts the stock market. *Journal of Computational Science,**2*(1), 1–8.

[CR14] Bouma, G. (2009). Normalized (pointwise) mutual information in collocation extraction. *Proceedings of the Biennial GSCL Conference, 2009* Normalized, 31–40.

[CR15] Breiman, L. (2001). Random forests. *Machine Learning,**45*(1), 5–32.

[CR16] Bussotti, J.-F., & Papotti, P. (2025). Refining attention for explainable and noise-robust fact-checking with transformers. In Christodoulopoulos, C., Chakraborty, T., Rose, C., and Peng, V., editors, *Proceedings of the 2025 Conference on Empirical Methods in Natural Language Processing*, pages 25487–25499, Suzhou, China. Association for Computational Linguistics.

[CR17] Cameron, M. P., Barrett, P., & Stewardson, B. (2016). Can social media predict election results? evidence from new zealand. *Journal of Political Marketing,**15*(4), 416–432.

[CR18] Cano-Marin, E., Mora-Cantallops, M., & Sanchez-Alonso, S. (2023). Twitter as a predictive system: A systematic literature review. *Journal of Business Research,**157*, Article 113561.

[CR19] Cecchini, M., Aytug, H., Koehler, G. J., & Pathak, P. (2010). Making words work: Using financial text as a predictor of financial events. *Decision Support Systems,**50*(1), 164–175.

[CR20] Church, K. W., & Hanks, P. (1990). Word association norms, mutual information, and lexicography. *Computational Linguistics,**16*(1), 22–29.

[CR21] Dangl, T., & Halling, M. (2012). Predictive regressions with time-varying coefficients. *Journal of Financial Economics,**106*(1), 157–181.

[CR22] Doumpos, M., Niklis, D., Zopounidis, C., & Andriosopoulos, K. (2015). Combining accounting data and a structural model for predicting credit ratings: Empirical evidence from european listed firms. *Journal of Banking & Finance,**50*, 599–607.

[CR23] Fernández-Gavilanes, M., Álvarez-López, T., Juncal-Martí­nez, J., Costa-Montenegro, E., & González-Castaño, F. J. (2016). Unsupervised method for sentiment analysis in online texts. *Expert Systems with Applications,**58*, 57–75.

[CR24] Ferreira, J. A., & Zwinderman, A. H. (2006). On the benjamini-hochberg method. *Annals of Statistics,**34*(4), 1827–1849.

[CR25] Figa-Talamanca, G., & Patacca, M. (2022). An explorative analysis of sentiment impact on s&p 500 components returns, volatility and downside risk. *Annals of Operations Research*.

[CR26] Friedl, M., & Brodley, C. (1997). Decision tree classification of land cover from remotely sensed data. *Remote Sensing of Environment,**61*(3), 399–409.

[CR27] Garcí­a, V., Marqués, A. I., and Sánchez, J. S. (2019). Exploring the synergetic effects of sample types on the performance of ensembles for credit risk and corporate bankruptcy prediction. *Information Fusion,**47*, 88–101.

[CR28] González-Fernández, M., & González-Velasco, C. (2020). An alternative approach to predicting bank credit risk in europe with google data. *Finance Research Letters,**35*, Article 101281.

[CR29] Hájek, P., & Michalak, K. (2013). Feature selection in corporate credit rating prediction. *Knowledge-Based Systems,**51*, 72–84.

[CR30] Hájek, P., & Olej, V. (2011). Credit rating modelling by kernel-based approaches with supervised and semi-supervised learning. *Neural Computing and Applications,**20*, 761–773.

[CR31] Hájek, P., Olej, V., & Procházka, O. (2017). Predicting corporate credit ratings using content analysis of annual reports - a naive bayesian network approach. In S. Feuerriegel & D. Neumann (Eds.), *Enterprise Applications, Markets and Services in the Finance Industry* (pp. 47–61). Cham. Springer International Publishing.

[CR32] Hand, D., & Till, R. (2001). A simple generalisation of the area under the roc curve for multiple class classification problems. *Machine Learning,**45*, 171–186.

[CR33] Hernandez Tinoco, M., & Wilson, N. (2013). Financial distress and bankruptcy prediction among listed companies using accounting, market and macroeconomic variables. *International Review of Financial Analysis,**30*, 394–419.

[CR34] Hillegeist, S., Keating, E., Cram, D., & Lundstedt, K. (2004). Assessing the probability of bankruptcy. *Review of Accounting Studies,**9*, 5–34.

[CR35] Hu, M., & Liu, B. (2004). Mining and summarizing customer reviews. In *Proceedings of the Tenth ACM SIGKDD International Conference on Knowledge Discovery and Data Mining*, KDD ’04, pages 168–177, New York, NY, USA. ACM.

[CR36] Huang, Z., Chen, H., Hsu, C.-J., Chen, W.-H., & Wu, S. (2004). Credit rating analysis with support vector machines and neural networks: a market comparative study. *Decision Support Systems,**37*(4), 543–558. Data mining for financial decision making.

[CR37] Hwang, R.-C., Cheng, K. F., & Lee, C.-F. (2009). On multiple-class prediction of issuer credit ratings. *Applied Stochastic Models in Business and Industry,**25*(5), 535–550.

[CR38] Hwang, R.-C., Chung, H., & Chu, C. (2010). Predicting issuer credit ratings using a semiparametric method. *Journal of Empirical Finance,**17*(1), 120–137.

[CR39] Jackson, R. H., & Wood, A. (2013). The performance of insolvency prediction and credit risk models in the uk: A comparative study. *The British Accounting Review,**45*(3), 183–202.

[CR40] Jones, S., Johnstone, D., & Wilson, R. (2015). An empirical evaluation of the performance of binary classifiers in the prediction of credit ratings changes. *Journal of Banking & Finance,**56*, 72–85.

[CR41] Jung, M. J., Naughton, J. P., Tahoun, A., & Wang, C. (2018). Do firms strategically disseminate? evidence from corporate use of social media. *The Accounting Review,**93*(4), 225–252.

[CR42] Kearney, C., & Liu, S. (2014). Textual sentiment in finance: A survey of methods and models. *International Review of Financial Analysis,**33*, 171–185.

[CR43] Kim, Y., & Sohn, S. Y. (2008). Random effects model for credit rating transitions. *European Journal of Operational Research,**184*(2), 561–573.

[CR44] Kolchyna, O., Souza, T. T. P., Treleaven, P., & Aste, T. (2015). *Twitter Sentiment Analysis: Lexicon Method, Machine Learning Method and Their Combination*.

[CR45] Kumar, K., & Bhattacharya, S. (2006). Artificial neural network vs linear discriminant analysis in credit ratings forecast. *Review of Accounting and Finance,**5*(3), 216–227.

[CR46] Lee, Y.-C. (2007). Application of support vector machines to corporate credit rating prediction. *Expert Systems with Applications,**33*(1), 67–74.

[CR47] Lerner, J., & Tirole, J. (2002). Some simple economics of open source. *The Journal of Industrial Economics,**50*(2), 197–234.

[CR48] Liaw, A., & Wiener, M. (2002). Classification and regression by randomforest. *R News,**2*(3), 18–22.

[CR49] Liebmann, M., Orlov, A. G., & Neumann, D. (2016). The tone of financial news and the perceptions of stock and cds traders. *International Review of Financial Analysis,**46*, 159–175.

[CR50] Liu, Y., Li, H., Guo, Y., Kong, C., Li, J., & Wang, S. (2022). Rethinking attention-model explainability through faithfulness violation test.

[CR51] Loughran, T., & Mcdonald, B. (2011). When is a liability not a liability? textual analysis, dictionaries, and 10-ks. *The Journal of Finance,**66*(1), 35–65.

[CR52] Matthies, A. B. (2013). Empirical research on corporate credit-ratings: A literature review. SFB 649 Discussion Paper 2013-003, Berlin.

[CR53] Merton, R. C. (1974). On the pricing of corporate debt: The risk structure of interest rates. *The Journal of Finance,**29*(2), 449–470.

[CR54] Nam, C., Kim, T., Park, N., & Lee, H. (2008). Bankruptcy prediction using a discrete-time duration model incorporating temporal and macroeconomic dependencies. *Journal of Forecasting,**27*, 493–506.

[CR55] Neely, M., Schouten, S. F., Bleeker, M., & Lucic, A. (2022). A song of (dis)agreement: Evaluating the evaluation of explainable artificial intelligence in natural language processing.

[CR56] Niresh, J. A., & Pratheepan, T. (2015). The application of altman’s z-score model in predicting bankruptcy: Evidence from the trading sector in sri lanka. *Forecasting Models eJournal*.

[CR57] Ohlson, J. A. (1980). Financial ratios and the probabilistic prediction of bankruptcy. *Journal of Accounting Research,**18*(1), 109–131.

[CR58] Oliveira, N., Cortez, P., & Areal, N. (2013). Some experiments on modeling stock market behavior using investor sentiment analysis and posting volume from twitter. In *Proceedings of the 3rd International Conference on Web Intelligence, Mining and Semantics*, WIMS ’13, pages 31:1–31:8, New York, NY, USA. ACM.

[CR59] Pang, D., Eichstaedt, J., Buffone, A., Slaff, B., Ruch, W., & Ungar, L. (2019). The language of character strengths: Predicting morally valued traits on social media. *Journal of Personality,**88*, 287–306. 31107975 10.1111/jopy.12491 PMC7065131

[CR60] Park, J. W., Nam, G., Tsang, A., & Lee, Y.-J. (2022). Firstborn ceos and credit ratings. *The British Accounting Review,**54*(4), Article 101083.

[CR61] Putra, S. G. P. (2018). Sme credit scoring using social media data. Master’s thesis, Delft University of Technology, Delft, the Netherlands.

[CR62] Qu, Y. (2008). Macro economic factors and probability of default. *European Journal of Economics, Finance and Administrative Sciences,**13*, 192–215.

[CR63] Ranco, G., Aleksovski, D., Caldarelli, G., Grcar, M., & Mozetic, I. (2015). The effects of twitter sentiment on stock price returns. *PLOS ONE,**10*(9), 1–21. 10.1371/journal.pone.0138441 PMC4577113 26390434

[CR64] Safavian, S. R., & Landgrebe, D. (1991). A survey of decision tree classifier methodology. *IEEE Transactions on Systems, Man, and Cybernetics,**21*(3), 660–674.

[CR65] Schmidt, C. W. (2012). Trending now: Using social media to predict and track disease outbreaks. *Environmental Health Perspectives,**120*(1), a30–a33. 22214548 10.1289/ehp.120-a30 PMC3261963

[CR66] Schwartz, H., Eichstaedt, J., Dziurzynski, L., Kern, M., Blanco, E., Kosinski, M., Stillwell, D., Seligman, M., & Ungar, L. (2013a). Toward personality insights from language exploration in social media. *2013 AAAI Spring Symposium. Association for the Advancement of Artificial Intelligence*.

[CR67] Schwartz, H. A., Eichstaedt, J. C., Kern, M. L., Dziurzynski, L., Ramones, S. M., Agrawal, M., Shah, A., Kosinski, M., Stillwell, D., Seligman, M. E. P., & Ungar, L. H. (2013). Personality, gender, and age in the language of social media: The open-vocabulary approach. *PLOS ONE,**8*(9), 1–16. 10.1371/journal.pone.0073791 PMC3783449 24086296

[CR68] Schwartz, H. A., Giorgi, S., Sap, M., Crutchley, P., Ungar, L., & Eichstaedt, J. (2017). DLATK: Differential language analysis ToolKit. In *Proceedings of the 2017 Conference on Empirical Methods in Natural Language Processing: System Demonstrations*, pages 55–60, Copenhagen, Denmark. Association for Computational Linguistics.

[CR69] Si, J., Mukherjee, A., Liu, B., Li, Q., Li, H., & Deng, X. (2013). Exploiting topic based twitter sentiment for stock prediction. In *Proceedings of the 51st Annual Meeting of the Association for Computational Linguistics (Volume 2: Short Papers)*, pages 24–29. Association for Computational Linguistics.

[CR70] Teplova, T., Kurkin, A., & Baklanova, V. (2023). Investor sentiment and the nft market: prediction and interpretation of daily nft sales volume. *Annals of Operations Research*, pages 1–25.

[CR71] Thissen, D., Steinberg, L., & Kuang, D. (2002). Quick and easy implementation of the benjamini-hochberg procedure for controlling the false positive rate in multiple comparisons. *Journal of Educational and Behavioral Statistics,**27*(1), 77–83.

[CR72] Uddin, M. S., Chi, G., Al Janabi, M. A. M., & Habib, T. (2020). Leveraging random forest in micro-enterprises credit risk modelling for accuracy and interpretability. *International Journal of Finance & Economics*, n/a(n/a).

[CR73] Wiegreffe, S., & Pinter, Y. (2019). Attention is not explanation. In Inui, K., Jiang, J., Ng, V., and Wan, X., editors, *Proceedings of the 2019 Conference on Empirical Methods in Natural Language Processing and the 9th International Joint Conference on Natural Language Processing (EMNLP-IJCNLP)*, pages 11–20, Hong Kong, China. Association for Computational Linguistics.

[CR74] Yang, S., Liu, Z., & Wang, X. (2020). News sentiment, credit spreads, and information asymmetry. *The North American Journal of Economics and Finance,**52*, Article 101179.

[CR75] Yuan, H., Lau, R. Y. K., Wong, M. C. S., & Li, C. (2018). Mining emotions of the public from social media for enhancing corporate credit rating. In *2018 IEEE 15th International Conference on e-Business Engineering (ICEBE)*, pages 25–30.

[CR76] Zamani, M., Buffone, A., & Schwartz, H. A. (2018). Predicting human trustfulness from facebook language. In *Proceedings of the Fifth Workshop on Computational Linguistics and Clinical Psychology: From Keyboard to Clinic*, pages 174–181. ACM.

[CR77] Zhao, Y., Shen, Y., & Huang, Y. (2019). Dmdp: A dynamic multi-source default probability prediction framework. *Data Science and Engineering,**4*, 3–13.

